# Tracking KP.2 SARS-CoV-2 Variant in India and the Clinical Profile of KP.2 Cases in Maharashtra, India

**DOI:** 10.7759/cureus.66057

**Published:** 2024-08-03

**Authors:** Rajesh P Karyakarte, Rashmita Das, Varsha Potdar, Bhakti Kulkarni, Marie Joy, Mansi Mishra, Jyoti Bhagat, Kalyani Jagarwal, Preeti Pawar, Damini More, Gilbert Chamy, Viswanathan DV, Sushma Yanamandra, Nyabom Taji, Jyoti Gurav, Suvarna Joshi

**Affiliations:** 1 Microbiology, Byramjee Jeejeebhoy Government Medical College & Sassoon General Hospitals, Pune, IND; 2 Infectious Disease, Indian Council of Medical Research-National Institute of Virology, Pune, IND; 3 Pediatrics, Byramjee Jeejeebhoy Government Medical College, Pune, IND; 4 Microbiology, B. J. Government Medical College & Sassoon General Hospitals, Pune, IND; 5 Integrated Disease Surveillance Program, Directorate of Health Services, Government of Maharashtra, Pune, IND

**Keywords:** clinical characteristics, covid-19, sars-cov-2, flirt variant, kp.2

## Abstract

Background: Following the emergence of the JN.1 SARS-CoV-2 variant, variants with key mutations in the spike protein, such as L455F, F456L, and R346T, were identified. In early January 2024, the KP.2 (JN.1.11.1.2) variant was first identified in clinical samples. Its increasing global prevalence has raised concerns over its transmission and clinical impact. The study investigates KP.2*’s (*indicates KP.2 and all its sub-lineages) spread and clinical severity in Maharashtra.

Methods: This study involved 5,173 Indian SARS-CoV-2 whole genome sequences with collection dates between November 1, 2023 and June 24, 2024. Lineage analysis of sequences was performed using Nextclade software (version 3.8.0). Telephonic interviews were conducted to confirm the demographic details and obtain clinical information on the KP.2* cases. The obtained data were recorded and analyzed using Microsoft® Excel (Microsoft Corporation, Redmond, WA).

Results: Among the 5,173 sequences analyzed, JN.1* appeared as the predominant lineage (65.96%, 3412/5173), followed by KP.2* (7.83%, 405/5173) and KP.1* (3.27%, 169/5173). In India, KP.2* was first detected on December 2, 2023, in Odisha. The majority of KP.2* sequences were from Maharashtra (248/405, 61.23%), followed by West Bengal (38/405, 9.38%), Gujarat (27/405, 6.67%), and Rajasthan (24/405, 5.93%). Maharashtra reported its first KP.2* sequences on January 24, 2024. The clinical study included 160 cases of the KP.2* variant from Maharashtra. Of these, 95.63% (153/160) presented with mild symptoms, such as fever (108/160, 67.50%), cold (87/160, 54.38%), cough (80/160, 50%), sore throat (44/160, 27.5%), body ache (43/160, 26.88%), and fatigue (42/160, 26.25%). About 33.13% (53/160) of the cases required institutional quarantine or hospitalization, with the rest managed at home. Among those hospitalized, 50.94% (27/53) received conservative treatment, while 49.06% (26/53) needed supplemental oxygen, steroids, or antiviral therapy. Regarding the vaccination status, 89.38% (143/160) of the cases had received at least one dose of the COVID-19 vaccine, whereas 10% (16/160) were unvaccinated, with the majority of the unvaccinated being children aged zero to nine years (7/16, 43.75%). The overall recovery rate for KP.2* cases was 99.38% (159/160), with only 0.62% (1/160) succumbing to the disease.

Conclusion: The KP.2 variant has become the dominant SARS-CoV-2 variant in India and Maharashtra. Despite the affected individuals experiencing mild symptoms, studies have shown lower neutralization titers and high infectivity due to FLiRT mutations, suggesting KP.2’s potential rise to global dominance.

## Introduction

The emergence and evolution of SARS-CoV-2 variants continue to challenge global public health efforts. In 2023, the BA.2.86 variant attracted considerable attention [1]. It was categorized as a variant under monitoring (VUM) by the World Health Organization (WHO) on August 17, 2023 [2]. By late 2023, attention had shifted to its descendant, JN.1 (BA.2.86.1), leading to increased worldwide cases [1]. Consequently, BA.2.86 and its descendant JN.1 were reclassified by the WHO as variants of interest (VOI) on November 21, 2023 [2], and December 20, 2023 [3], respectively.

Before the emergence of JN.1* (*indicates JN.1 and all its sub-lineages), the most prevalent variants were XBB and its sub-lineages. As the XBB evolved, several variants with mutations at key sites in the spike (S) protein - L455 and F456 - have been identified. During the same period, the so-called FLip variants appeared, characterized by spike (S) protein mutations - L455F and F456L - in the backbone of XBB.1.5, hence named “FLip”. Since the emergence of JN.1, these mutation sites in the S protein have remained hotspots, leading to the appearance of the so-called SLip variants. The “SLip” variants combine the JN.1 spike protein (possessing L455S) with the F456L mutation. Therefore, it was named “SLip”. More recently, the so-called FLiRT variant emerged, featuring an additional R346T mutation on the “SLip” backbone, thus named “FLiRT”. The KP.2 (JN.1.11.1.2) SARS-CoV-2 variant, a descendant of the JN.1 variant, is an example of the “FLiRT” variant [4]. It was officially designated as KP.2 on May 3, 2024. The KP.2 variant is characterized by three defining FLiRT mutations in the S protein - F456L, R346T, and V1104L [5]. Due to its increasing global prevalence, the WHO has classified KP.2 and KP.3 as VUM [6]. The earliest documented sample of the KP.2 variant was collected on January 2, 2024 [5]. Globally, KP.2 accounted for 22.7% of sequences in Week 21, 2024 [6].

Considering the increasing prevalence of the KP.2 variant globally, there is a pressing need to understand its impact at a regional level. Maharashtra, a state in India with a significant population density and diverse demographics, offers a valuable case study for observing the behavior of this variant. Therefore, this study aimed to understand the appearance, prevalence, and clinical outcomes of the KP.2 variant within Maharashtra.

## Materials and methods

This study is a part of the sequencing efforts in Maharashtra under the Indian SARS-CoV-2 Genomics (INSACOG) consortium to investigate the evolutionary patterns of SARS-CoV-2.

Lineage analysis of SARS-CoV-2 whole genome sequences in Maharashtra

To trace the first appearance and the spread of the KP.2 SARS-CoV-2 variant in Maharashtra, whole genome sequences of the virus, collected between November 1, 2023, and June 24, 2024, were analyzed. These sequences, submitted by various sequencing laboratories across states and union territories, were downloaded with permission from the Indian Biological Data Centre (IBDC) database [7]. Only entries with complete metadata, including geographic locations and sample collection dates, were included in the study. The sequences used can be found in Tables 4-5 (Appendices). Lineage and clade analysis were performed using Nextclade software (version 3.8.0).

Collection of demographic and clinical data of SARS-CoV-2 positive cases in Maharashtra

Demographic details, including age, sex, area of residence, contact number, and dates of sample collection and testing, were obtained from the metadata provided to sequencing laboratories by real-time PCR (RT-PCR) testing centers. Telephonic interviews were conducted with each patient to verify demographic details and gather clinical information. Information collected included symptoms, type of isolation, hospitalization, oxygen requirements, treatments, and vaccination status. The questionnaire template used in the study is available in Tables 4-5 (Appendices). Patients who did not consent to disclose their clinical history during the interview were noted and excluded from the study.

Daily records were collected from the State’s District Health Services Department, including daily SARS-CoV-2 testing and test positivity rates from November 1, 2023 and June 24, 2024. This data was used to assess any surge in COVID-19 cases associated with the appearance of the KP.2* SARS-CoV-2 variant in Maharashtra. Additionally, SARS-CoV-2 test positivity rates from clinical samples were compared with viral loads in wastewater from the Pune Municipal Corporation. To establish the correlation, the publicly accessible dashboard describing SARS-CoV-2 variants in Pune’s wastewater sampling was studied [8].

Statistical analysis

All demographic and clinical data were recorded and analyzed using Microsoft® Excel, and analysis was performed using Microsoft® Excel. The continuous variables were presented as the median and interquartile range (IQR). The categorical variables were presented as numbers and percentages.

## Results

Distribution of SARS-CoV-2 lineages in Maharashtra and India

A total of 5,173 downloaded sequences were included in the study. Following Nextclade Pangolin nomenclature, 129 different lineages were identified between November 1, 2023, and June 24, 2024. JN.1* was the most common lineage (65.96%), followed by KP.2* (7.83%) and KP.1* (3.27%) (Table 1).

**Table 1:** SARS-CoV-2 variant distribution in India Based on sequences submitted to the Indian Biological Data Centre (IBDC)

Pangolin Lineage		Nextclade Clade	Count	
BA.1	BA.1.1	21K	1	1 (0.02%)
BA.2*	BA.2	24A	155	156 (3.02%)
	BA.2.10.1	23F	1	
BA.2.38	BA.2.38	21L	1	1 (0.02%)
BA.2.86*	BA.2.86	23I	2	130 (2.51%)
	BA.2.86.1		28	
	KU.1	24A	7	
	KU.2		19	
	KZ.1		3	
	KZ.1.1		11	
	LB.1		37	
	LC.1		23	
JN.1*	JN.1		1045	3412 (65.96%)
	JN.1.1		1194	
	JN.1.1.1		2	
	JN.1.1.3		2	
	JN.1.1.5		11	
	JN.1.1.6		22	
	JN.1.1.7		6	
	JN.1.10		4	
	JN.1.11	24B	421	
	JN.1.11.1		143	
	JN.1.13	24A	1	
	JN.1.15		1	
	JN.1.16		6	
	JN.1.16.1		5	
	JN.1.18		72	
	JN.1.18.1		3	
	JN.1.19		2	
	JN.1.2		3	
	JN.1.20		3	
	JN.1.22		10	
	JN.1.25		18	
	JN.1.25.1		32	
	JN.1.28		3	
	JN.1.28.1		1	
	JN.1.29		1	
	JN.1.3		4	
	JN.1.30		109	
	JN.1.30.1		35	
	JN.1.32		9	
	JN.1.33		1	
	JN.1.37		1	
	JN.1.38		2	
	JN.1.39		15	
	JN.1.4		34	
	JN.1.4.2		1	
	JN.1.4.4		1	
	JN.1.4.5		21	
	JN.1.4.7		1	
	JN.1.40		1	
	JN.1.43.1		1	
	JN.1.44		1	
	JN.1.47		1	
	JN.1.48		100	
	JN.1.48.1		11	
	JN.1.5		22	
	JN.1.6		3	
	JN.1.6.1		1	
	JN.1.7.2		1	
	JN.1.8		7	
	JN.1.8.3		8	
	JN.1.9		2	
	JN.1.9.2		8	
JN.12	JN.12	23I	1	1 (0.02%)
JN.2	JN.2		3	3 (0.06%)
JN.6	JN.6		1	1 (0.02%)
KP.1*	KP.1	24B	35	169 (3.27%)
	KP.1.1		98	
	KP.1.1.1		30	
	KP.1.2		6	
KP.2*	KP.2		299	405 (7.83%)
	KP.2.1		2	
	KP.2.2		9	
	KP.2.3		95	
KP.3	KP.3		69	69 (1.33%)
KP.4*	KP.4		15	105 (2.03%)
	KP.4.1		39	
	KP.4.2		51	
XBB*	XBB.1	23B	5	7 (0.14%)
	XBB.1.28.1	23F	1	
	XBB.1.41.1		1	
XBB.1.5*	GK.1.1	23G	1	5 (0.1%)
	JD.1.1	23A	2	
	JD.1.1.1		1	
	XBB.1.5.45		1	
XBB.1.9*	EG.5.1	23F	1	29 (0.56%)
	EG.5.1.1	23H	2	
	EG.5.1.8	23F	1	
	FL.1.5.1	23D	1	
	FL.13.2		1	
	FL.13.4		1	
	FL.4.8		1	
	HK.3	23H	2	
	HK.3.1		2	
	HV.1	23F	11	
	HV.1.11		3	
	JG.3		2	
	JG.3.2		1	
XBB.2.3*	GE.1	23E	18	52 (1.01%)
	GE.1.1		1	
	GJ.1		3	
	GJ.1.1		1	
	GJ.3		1	
	GJ.6		1	
	GS.1		1	
	GZ.1		1	
	JE.1.1.1		1	
	JY.1		11	
	JY.1.1		6	
	XBB.2.3		3	
	XBB.2.3.19		1	
	XBB.2.3.3		3	
XBB.1.16*	FU.3	23B	1	78 (1.51%)
	JF.1.1		1	
	XBB.1.16		37	
	XBB.1.16.1		5	
	XBB.1.16.11		16	
	XBB.1.16.17		9	
	XBB.1.16.18		1	
	XBB.1.16.19		1	
	XBB.1.16.2		2	
	XBB.1.16.20		1	
	XBB.1.16.24		4	
XBB.1.19*	GW.5.1.1	22F	1	3 (0.06%)
	GW.5.3.1		2	
Recombinant	XDA	Recombinant	7	28 (0.54%)
	XDD		2	
	XDK.1		19	
Unassigned			518	518 (10.01%)
Grand Total			5173	5173 (100%)

**Figure 1:**
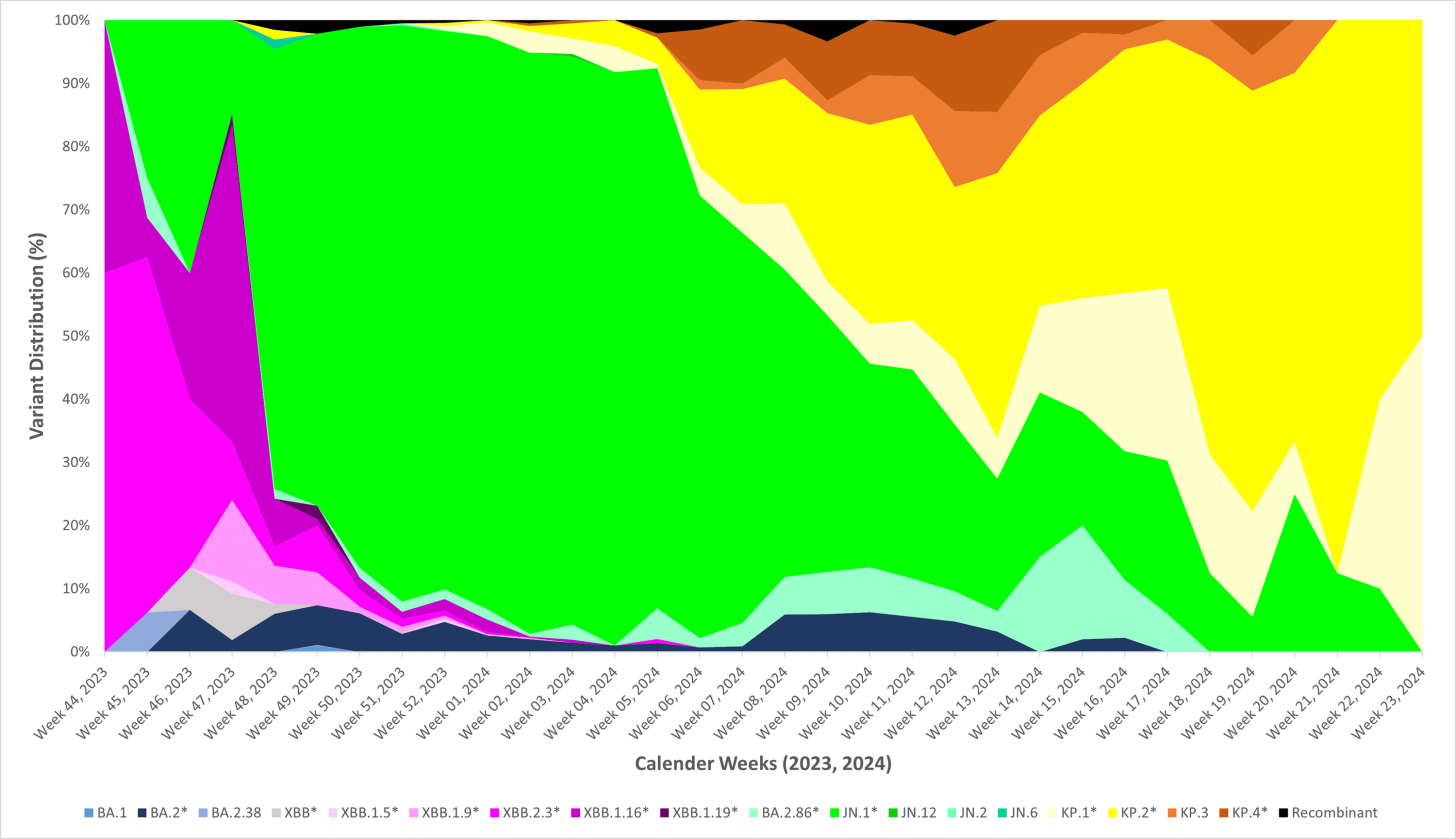
Temporal distribution of SARS-CoV-2 variants in India Based on sequences submitted to the Indian Biological Data Centre (IBDC) Image credits: Rashmita Das

**Figure 2:**
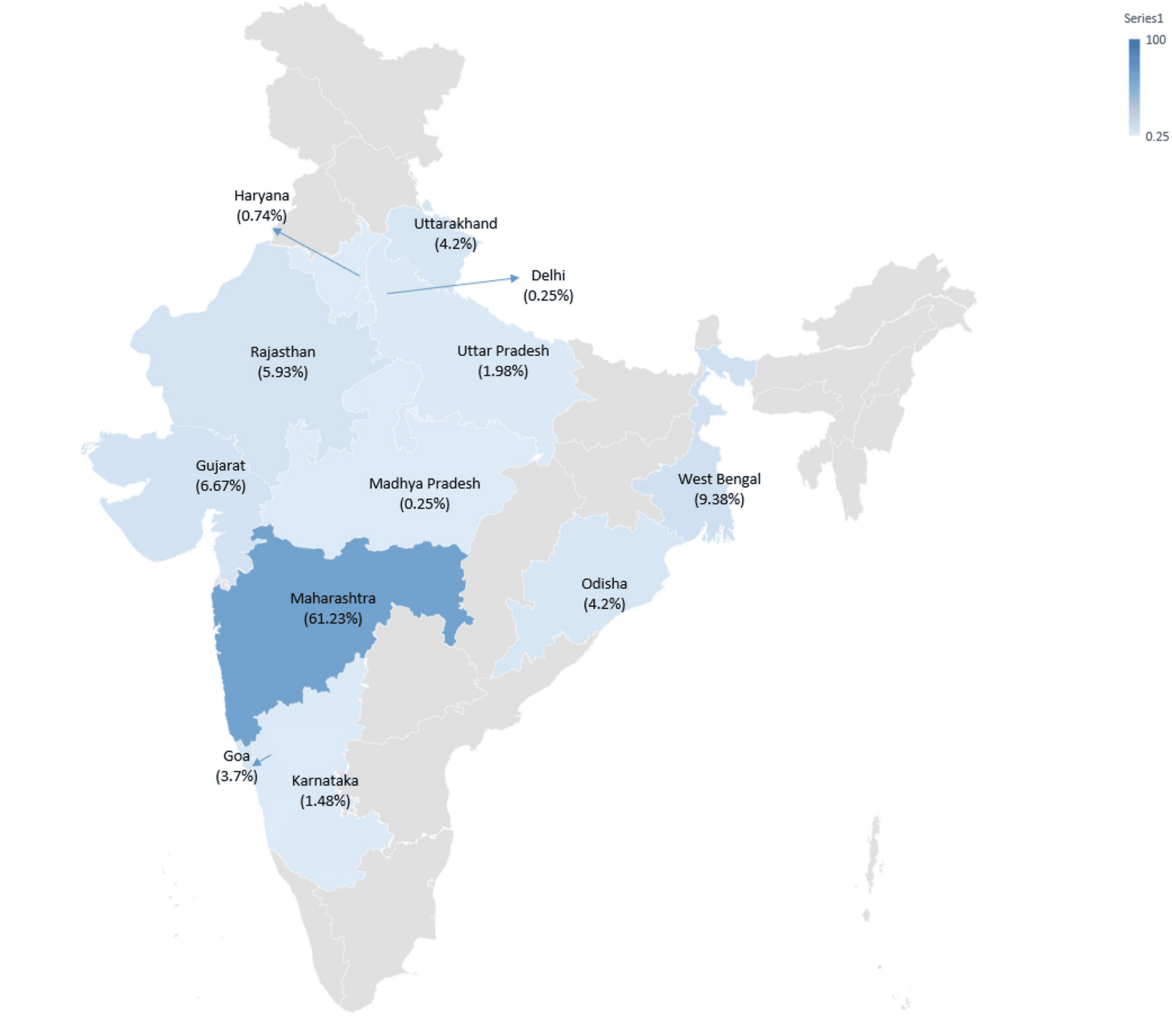
Geographic distribution of the KP.2* SARS-CoV-2 variant in India Based on sequences submitted to the Indian Biological Data Centre (IBDC) Image credits: Rashmita Das

In India, the distribution of SARS-CoV-2 variants over time indicates that the JN.1 variant and its associated lineages remained prevalent until early 2024 (Figure 1) The KP.2* variant was first identified in Indian sequences on December 2, 2023 (Week 48, 2023), in Odisha. By mid-2024, KP.2* had become the most widespread variant nationwide. Its prevalence rose from 0.56% in Week 52, 2023, to 18.18% in Week 7, 2024. Among all sequences submitted by different states, 8.70% were of the KP.2* variant. Maharashtra accounted for the highest proportion of KP.2* sequences (248/405, 61.23%), followed by West Bengal (38/405, 9.38%), Gujarat (27/405, 6.67%), and Rajasthan (24/405, 5.93%) (Figure 2).

**Figure 3:**
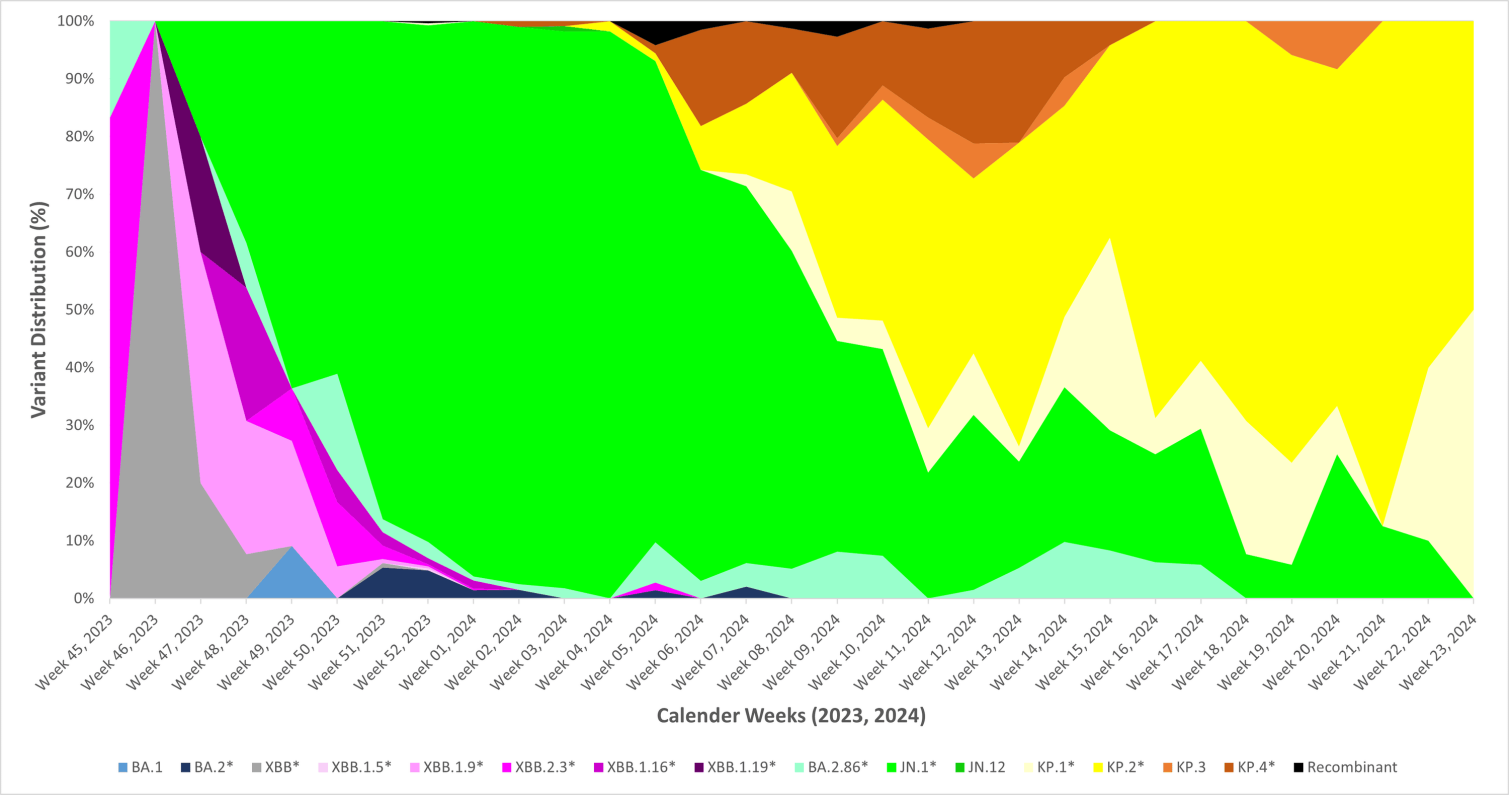
Temporal distribution of SARS-CoV-2 variants in Maharashtra Based on sequences submitted to the Indian Biological Data Centre (IBDC) Image credits: Rashmita Das

Figure 3 illustrates the weekly distribution of SARS-CoV-2 variants in Maharashtra over six months, reflecting the national trend. The KP.2* variant was first identified on January 24, 2024 (Week 4, 2024), and its prevalence increased from 1.79% in Week 4, 2024, to 50% by Week 11, 2024.

SARS-CoV-2 testing and positivity trends in Maharashtra

**Figure 4:**
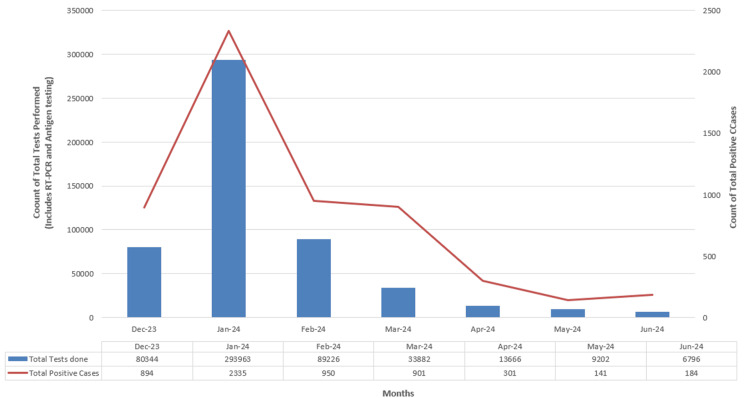
Weekly tests performed versus weekly count of positive cases in Maharashtra over six months Image credits: Rashmita Das

Figure 4 provides an overview of SARS-CoV-2 testing in Maharashtra, depicting the total number of tests conducted (including RT-PCR and antigen testing) and the positivity rate over six months. January 2024 saw the highest testing and positive cases, though the positivity rate was relatively low at 0.79%. From February 2024 onward, both testing and positive cases gradually declined. The positivity rates varied, with significant increases in March (2.66%) and June 2024 (2.20%). The positivity rate of 2.71% occurred in June 2024, despite the lowest testing, suggesting a higher proportion of positive cases among those tested.

Trends in SARS-CoV-2 positivity in Pune Municipal Corporation’s wastewater

**Figure 5:**
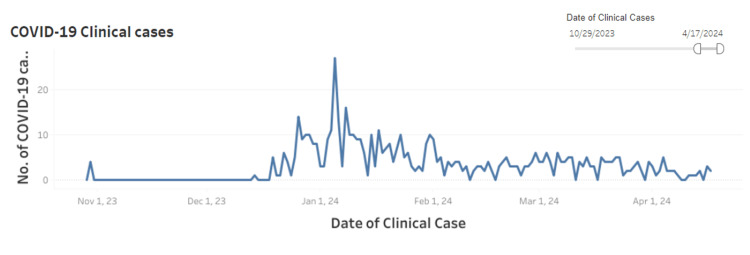
Trends in COVID-19 clinical cases in Pune city Figure reproduced from the image available at the wastewater surveillance dashboard for infectious diseases (COVID-19, H1N1, H3N2, Influenza-A) – the Pune Knowledge Cluster (PKC) [8].

Figure 5 displays the trends in COVID-19 clinical cases in Pune over six months from November 1, 2023 to April 17, 2024. A notable peak is observed in early January 2024, reflecting similar trends observed across Maharashtra. Following the peak, the number of cases gradually declined, settling into a relatively steady level from late January to April 2024.

**Figure 6:**
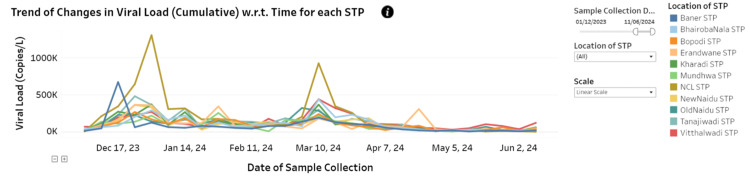
Trends in viral load variations in wastewaters across Pune city Figure reproduced from the image available at the wastewater surveillance dashboard for infectious diseases (COVID-19, H1N1, H3N2, Influenza-A) – the Pune Knowledge Cluster (PKC) [8]. STP: sewage treatment plant

Figure 6 indicates the cumulative viral load (in copies per liter) across different sewage treatment plants (STPs) in Pune city over the six-month period. Notable peaks in viral load were observed in several STPs in December 2023 and March 2024, suggesting a widespread increase in viral load in the city. This aligns with the observed trends in COVID-19 case positivity within the city during the same timeframe.

**Figure 7:**
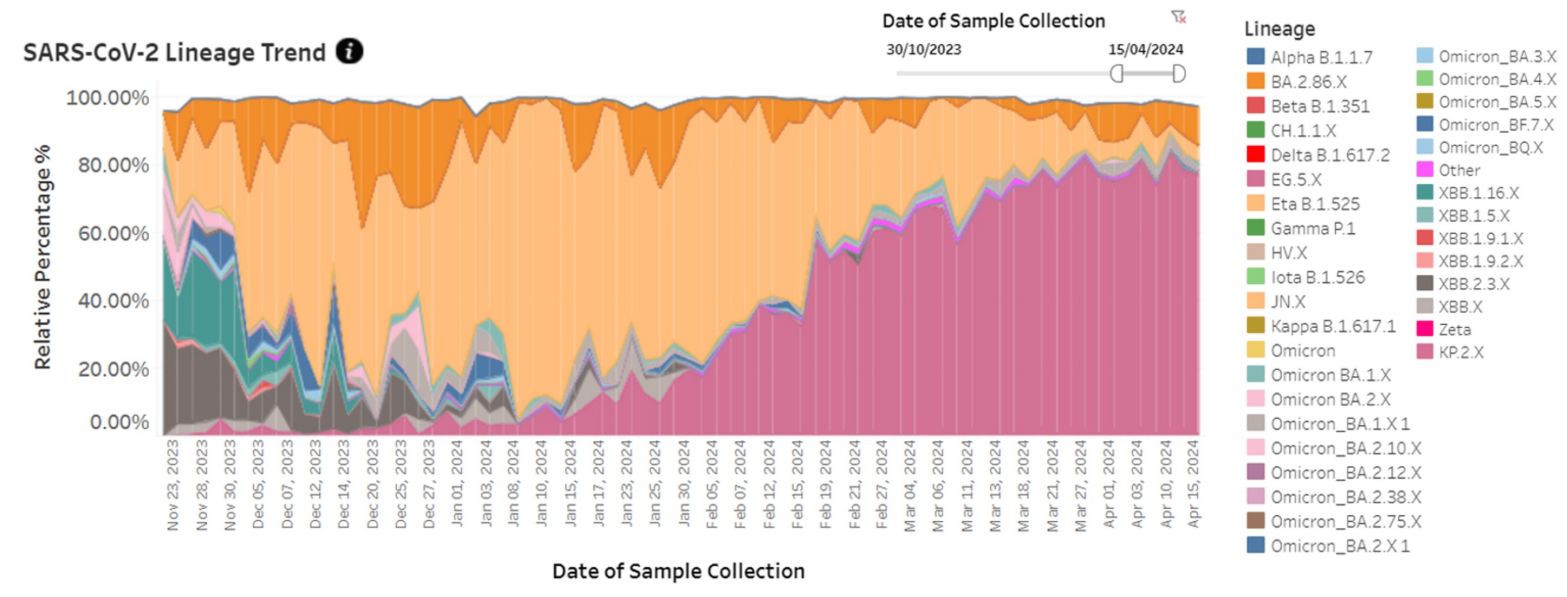
Trends in SARS-CoV-2 lineages detected in wastewaters across Pune city Figure reproduced from the image available at the wastewater surveillance dashboard for infectious diseases (COVID-19, H1N1, H3N2, Influenza-A) – the Pune Knowledge Cluster (PKC) [8]

Figure 7 illustrates the prevalence of different SARS-CoV-2 variants in wastewater samples from Pune city over the six-month period. Initially, in November and December 2023, the JN.X and BA.2.86.X variants were the most dominant variants. Subsequently, a visible shift occurred in the variant distribution, with the KP.2.X variant becoming the dominant lineage during the latter half of the period. The KP.2.X variant first appeared in the wastewater data around late November 2023.

Demographic and clinical characteristics of KP.2* SARS-CoV-2 variant in Maharashtra

Complete metadata was available for 274 cases included in the demographic study (Table 2). Among these 274 participants, 52.19% were male and 47.81% were female. The median age of the study population was 42 (IQR: 25-63) years. The largest age group comprised individuals aged 60 years and above, making up 30.66% of the study population. Most participants were from Pune (54.01%), followed by Thane (32.12%).

**Table 2:** Demographic characteristics of KP.2* SARS-CoV-2 variant in Maharashtra

Demographic Characteristics	Count (%)
Gender-wise distribution	
Male	143 (52.19%)
Female	131 (47.81%)
Age-wise distribution (years)	
0-9	15 (5.48%)
10-19	24 (8.76%)
20-29	43 (15.69%)
30-39	41 (14.96%)
40-49	37 (13.50%)
50-59	30 (10.95%)
60 and above	84 (30.66%)
Area-wise distribution in Maharashtra	
Ahmednagar	1 (0.36%)
Akola	1 (0.36%)
Amravati	11 (4.01%)
Chhatrapati Sambhajinagar	3 (1.09%)
Kolhapur	5 (1.82%)
Mumbai	2 (0.73%)
Nagpur	1 (0.36%)
Nashik	2 (0.73%)
Pune	148 (54.01%)
Sangli	1 (0.36%)
Satara	1 (0.36%)
Solapur	10 (3.65%)
Thane	88 (32.12%)

From this subset of 274 cases, attempts to contact 59 (21.53%) participants were unsuccessful due to non-response to calls, 33 (12.04%) had invalid numbers, and 22 (8.03%) participants declined participation in the study. Therefore, 160 (58.39%) participants were successfully contacted and included in the clinical research. Table 3 summarizes the clinical characteristics, vaccination status, and outcome of KP.2* cases in Maharashtra. Most KP.2* cases had symptomatic disease (95.63%) with mild symptoms. The most common symptoms were fever (67.50%), cold (54.38%), cough (50%), sore throat (27.5%), body ache (26.88%) and fatigue (26.25%). Underlying comorbid conditions, either alone or in combination, were reported in 25.62% of cases, with hypertension (65%), diabetes mellitus (42.50%), and chronic obstructive pulmonary disease (17.50%) being the most common conditions. Regarding the vaccination status, 89.38% (143 out of 160) of cases received at least one dose of the COVID-19 vaccine, while 10% (16 out of 160) were not vaccinated. Most unvaccinated individuals were children aged 0 to 9 years (7 out of 16, 43.75%) and children aged 10-19 years (4 out of 16, 25%).

Of all the cases, 33.13% (53 out of 160) required hospitalization. Nearly all the home-isolated patients (91.59%, 98 out of 107) received conservative treatment. In contrast, hospitalized patients often received conservative care (27 out of 53, 50.94%) or required supplemental oxygen/steroids/antiviral therapy (26 out of 53, 49.06%). The recovery rate was high; out of 160 cases, 99.38% of cases (159 out of 160) recovered from the disease, while 0.62% (1 out of 160) succumbed.

**Table 3:** Clinical characteristics of KP.2* SARS-CoV-2 variant in Maharashtra

Clinical Characteristics	Count (%)
History of previous infection	
Yes	22 (13.75%)
No	138 (86.25%)
Presence of comorbidities	
Present	41 (25.62%)
Hypertension (HTN)	14 (34.15%)
Diabetes mellitus (DM)	6 (14.63%)
Underlying heart disease	2 (4.88%)
Chronic obstructive pulmonary disease (COPD)	3 (7.32%)
Thyroid dysfunction	1 (2.44%)
HTN + DM	9 (21.95%)
DM + COPD	1 (2.44%)
HTN + COPD	1 (2.44%)
HTN + COPD + thyroid dysfunction	1 (2.44%)
HTN + DM + cerebrovascular attack	1 (2.44%)
COPD + malignancy	1 (2.44%)
Parkinson’s disease	1 (2.44%)
Absent	119 (74.38%)
Vaccination status	
Vaccinated	143 (89.38%)
One dose	9 (6.29%)
Two doses	86 (60.14%)
Booster dose (precautionary dose)	48 (33.57%)
Not vaccinated	16 (10%)
Data not available	1 (0.62%)
Symptom status	
Symptomatic	153 (95.63%)
Asymptomatic	7 (4.37%)
Presenting symptoms	
Fever	108 (65.50%)
Cold	87 (54.38%)
Cough	80 (50%)
Sore throat	44 (27.50%)
Body ache	43 (26.88%)
Fatigue	42 (26.25%)
Breathlessness	19 (11.88%)
Headache	14 (8.75%)
Vomiting	12 (7.50%)
Loss of taste	5 (3.13%)
Loss of smell	3 (1.88%)
Diarrhea	3 (1.88%)
Type of isolation	
Home isolation	107 (66.87%)
Hospital isolation/hospitalization	53 (33.13%)
Type of treatment received	
No treatment	8 (5.0%)
Symptomatic treatment (antipyretics, antihistaminic, multivitamins)	125 (78.13%)
Oxygen therapy	12 (7.50%)
Invasive	1 (8.33%)
Non-invasive	11 (91.67%)
Antiviral treatment	8 (5.0%)
Steroid	1 (0.63%)
Antiviral treatment + oxygen therapy	4 (2.50%)
Antiviral treatment + oxygen therapy + steroid	2 (1.25%)
Outcome of disease	
Alive	159 (99.38%)
Dead	1 (0.62%)

## Discussion

JN.1 remains the most frequently reported VOI globally, though its prevalence has declined from 56% in Week 18 to 47.1% in Week 21. Conversely, its descendant lineages, KP.2 and KP.3, have shown increasing prevalence, rising to 22.7% and 22.4% in Week 21 from 14.6% and 13% in Week 18, respectively [6]. Early analysis of KP. 2’s relative effective reproductive number (Re) using sequences from the USA, UK, and Canada indicated that the KP.2 Re exceeded that of JN.1 by 1.22 times in the USA, 1.32 times in the UK, and 1.26 times in Canada. Also, the Re of KP.2.3 (a sub-lineage of KP.2) was higher than that of KP.2. KP. 2’s three substitutions in the S protein compared to JN.1 potentially contributed to its increased viral fitness. This analysis predicted KP.2’s rise to global dominance, a position it reached in the UK by early April 2024 (prevalence of 20%) [9]. The KP.2* variant currently shows a global relative growth advantage of 30% (confidence interval 29-30%) [10]. Similarly, KP.2*’s relative growth advantage in India is 22% (confidence interval 19-25%) [11].

Further, a study by Kaku et al. supports the findings on the enhanced immune evasion and increased reproductive number (Re) of KP.2. They showed that KP.2 exhibited greater resistance to neutralization than the parental JN.1 [9]. Neutralization titers against KP.2.3 were 2.0-fold to 2.9-fold lower than those for JN.1 [12]. Another study investigated the JN.1-derived subvariants for their susceptibility to neutralization by sera from infected/vaccinated individuals, as well as monoclonal antibodies (Mabs). The study found that the three FLiRT mutations - L455S, F456L, and R346T - altered the epitopes targeted by therapeutic Mabs, reducing the sensitivity to neutralization by sera and Mabs [4]. Notably, KP.2.3 displayed higher pseudovirus infectivity and stronger immune resistance than KP.2. This highlights the crucial role of Ser31del mutation in enhancing infectivity and immune evasion capabilities among variants having the said mutation [12].

Kaku et al. also found that the infectivity of the KP.2 pseudovirus in human HOS-ACE2/TMPRSS2 cells was significantly lower than JN.1 [12]. Homology modeling indicated that the L455S and F456L mutations reduce the binding of spike protein to ACE2 receptors. However, the additional R346T mutation in the FLiRT and KP.2 variants strengthened the conformational stability of the receptor-binding motif, counteracting the effects of L455S and F456L mutations [4]. The R346T mutation increased receptor-binding affinity by approximately 50% and pseudovirus infectivity by around 40% [13].

The study highlights significant fluctuations in SARS-CoV-2 clinical testing and the positivity rates. January 2024 experienced the highest volume of tests and confirmed cases, yet the positivity rate remained comparatively low. Conversely, June 2024 saw the lowest testing volume but the highest positivity rate, suggesting that these figures may not accurately represent the actual disease burden within the community. The WHO has also indicated that trends in reported cases and deaths should be interpreted cautiously, as factors like reduced testing, sequencing, and reporting delays can distort actual figures across various regions [6]. Additionally, the data from Pune city, which had the most KP.2 cases in this study, shows a lower clinical detection of positive cases from February to April 2024 (Figure 5). However, analysis of wastewater samples from the Pune metropolitan region revealed that by April 2024, the KP.2.X variant had appeared as the predominant variant in wastewater (Figure 7) [8]. Also, the global wastewater surveillance data suggests that clinical testing might underreport the actual disease burden by approximately 2-19 times [6]. Therefore, relying solely on case-based surveillance may not suffice for the timely identification of novel variants. Broader, population-level surveillance strategies, particularly wastewater-based surveillance, are essential.

The study tracks the appearance and spread of the KP.2 variant in Maharashtra and India and provides a comprehensive description of the clinical characteristics associated with the variant. However, it suffers from a few limitations. Primarily, the study’s findings rely on data from individuals who underwent COVID-19 testing, had their samples sequenced and consented to participate in the study. This means the results may not fully capture the true extent of the disease. As the study focuses solely on data from Maharashtra, its applicability to other regions and populations with different demographic profiles may be limited. Also, the clinical data were collected through telephonic interviews, which could introduce recall bias in the study. Therefore, further studies must be conducted to improve accuracy and representativeness to include broader geographic areas and populations.

## Conclusions

The KP.2* variant has emerged as the dominant SARS-CoV-2 variant in India, causing symptoms such as fever, cold, cough, sore throat, body ache, and fatigue. Although these symptoms were generally not severe, the variant’s increased transmissibility and ability to evade immune responses underscore the virus's ongoing adaptability. Our study highlights the critical need for genomic surveillance based on clinical cases. With declining active surveillance SARS-CoV-2 testing rates, innovative surveillance methods like wastewater monitoring help to accurately assess the true burden of COVID-19. The study underscores that both of these epidemiological methods enable the public health authorities to forecast, mitigate, and prepare for potential outbreaks more effectively.

## Appendices

**Table 4:** IDs of the sequences used in the study Downloaded from the Indian Biological Data Centre (IBDC), Regional Centre for Biotechnology, Department of Biotechnology, Ministry of Science and Technology, Government of India, with their permission.

INCOV288208
INCOV288247
INCOV288218
INCOV288355
INCOV288860
INCOV287996
INCOV288599
INCOV288255
INCOV288416
INCOV288598
INCOV288638
INCOV288639
INCOV288640
INCOV292259
INCOV288631
INCOV290091
INCOV288151
INCOV290106
INCOV288016
INCOV288256
INCOV289069
INCOV288577
INCOV290092
INCOV288182
INCOV292258
INCOV288002
INCOV288033
INCOV288034
INCOV288183
INCOV288127
INCOV288186
INCOV288574
INCOV288626
INCOV292256
INCOV292257
INCOV288009
INCOV288356
INCOV288578
INCOV292255
INCOV288006
INCOV288013
INCOV288128
INCOV288185
INCOV288601
INCOV288602
INCOV288129
INCOV288184
INCOV288209
INCOV288210
INCOV288474
INCOV288489
INCOV288575
INCOV294047
INCOV294048
INCOV294049
INCOV294050
INCOV294051
INCOV294052
INCOV294053
INCOV294054
INCOV294055
INCOV294056
INCOV294057
INCOV294058
INCOV294059
INCOV294060
INCOV294061
INCOV294062
INCOV294063
INCOV294064
INCOV294065
INCOV294066
INCOV294067
INCOV294068
INCOV294069
INCOV294070
INCOV294071
INCOV294072
INCOV288248
INCOV288462
INCOV288570
INCOV288603
INCOV288861
INCOV291336
INCOV288130
INCOV288131
INCOV288627
INCOV289137
INCOV292253
INCOV292254
INCOV288234
INCOV288235
INCOV288463
INCOV288569
INCOV288357
INCOV290093
INCOV292487
INCOV288231
INCOV288507
INCOV288508
INCOV288509
INCOV288272
INCOV288286
INCOV288348
INCOV288475
INCOV288505
INCOV288506
INCOV288571
INCOV288572
INCOV288573
INCOV288604
INCOV288605
INCOV288628
INCOV289077
INCOV288132
INCOV288238
INCOV288239
INCOV288358
INCOV288476
INCOV288477
INCOV288478
INCOV288482
INCOV288483
INCOV288495
INCOV288576
INCOV292251
INCOV288133
INCOV288134
INCOV288298
INCOV288313
INCOV288331
INCOV288333
INCOV288492
INCOV288493
INCOV288494
INCOV288590
INCOV288629
INCOV288681
INCOV288682
INCOV288791
INCOV288792
INCOV288812
INCOV289059
INCOV289078
INCOV289079
INCOV289663
INCOV292324
INCOV288135
INCOV288152
INCOV288219
INCOV288301
INCOV288347
INCOV288582
INCOV288606
INCOV288793
INCOV289080
INCOV289700
INCOV291202
INCOV291809
INCOV291810
INCOV291812
INCOV291817
INCOV292325
INCOV292326
INCOV288299
INCOV288300
INCOV288336
INCOV288341
INCOV288346
INCOV288490
INCOV288491
INCOV288580
INCOV288592
INCOV292327
INCOV292328
INCOV288136
INCOV288211
INCOV288214
INCOV288240
INCOV288335
INCOV288421
INCOV288499
INCOV288501
INCOV288535
INCOV288583
INCOV288617
INCOV288794
INCOV289085
INCOV292488
INCOV292489
INCOV288137
INCOV288215
INCOV288220
INCOV288337
INCOV288479
INCOV288480
INCOV288498
INCOV288500
INCOV288608
INCOV288610
INCOV288611
INCOV288795
INCOV290017
INCOV290107
INCOV290108
INCOV292329
INCOV292330
INCOV288138
INCOV288221
INCOV288292
INCOV288297
INCOV288330
INCOV288359
INCOV288536
INCOV288584
INCOV288585
INCOV288607
INCOV288796
INCOV289664
INCOV290074
INCOV288139
INCOV288217
INCOV288222
INCOV288257
INCOV288293
INCOV288294
INCOV288295
INCOV288296
INCOV288360
INCOV288460
INCOV288496
INCOV288497
INCOV288504
INCOV288609
INCOV288630
INCOV288683
INCOV288684
INCOV288685
INCOV289127
INCOV289587
INCOV291384
INCOV292490
INCOV288140
INCOV288141
INCOV288212
INCOV288213
INCOV288223
INCOV288261
INCOV288262
INCOV288361
INCOV288362
INCOV288579
INCOV288586
INCOV288587
INCOV288588
INCOV288797
INCOV288142
INCOV288241
INCOV288319
INCOV288342
INCOV288363
INCOV288502
INCOV288503
INCOV288581
INCOV288589
INCOV288798
INCOV288799
INCOV288800
INCOV289083
INCOV289739
INCOV290025
INCOV290094
INCOV291807
INCOV288216
INCOV288290
INCOV288291
INCOV288320
INCOV288339
INCOV288340
INCOV288510
INCOV288537
INCOV289631
INCOV288143
INCOV288144
INCOV288224
INCOV288258
INCOV288267
INCOV288268
INCOV288269
INCOV288289
INCOV288321
INCOV288322
INCOV288349
INCOV288350
INCOV288364
INCOV288538
INCOV288539
INCOV288612
INCOV288635
INCOV288686
INCOV288801
INCOV288802
INCOV288803
INCOV288804
INCOV289081
INCOV289591
INCOV289607
INCOV289690
INCOV289745
INCOV289767
INCOV289776
INCOV290095
INCOV291071
INCOV292250
INCOV288145
INCOV288146
INCOV288147
INCOV288148
INCOV288149
INCOV288150
INCOV288259
INCOV288260
INCOV288270
INCOV288271
INCOV288287
INCOV288288
INCOV288312
INCOV288323
INCOV288324
INCOV288329
INCOV288343
INCOV288390
INCOV288485
INCOV288486
INCOV288488
INCOV288514
INCOV288591
INCOV288613
INCOV288805
INCOV288806
INCOV289068
INCOV289564
INCOV289574
INCOV289576
INCOV289581
INCOV289590
INCOV290070
INCOV290071
INCOV290099
INCOV290113
INCOV290114
INCOV290128
INCOV290946
INCOV288225
INCOV288242
INCOV288283
INCOV288284
INCOV288285
INCOV288325
INCOV288326
INCOV288344
INCOV288423
INCOV288424
INCOV288464
INCOV288481
INCOV288487
INCOV288511
INCOV288512
INCOV288513
INCOV288515
INCOV288618
INCOV288687
INCOV288688
INCOV288710
INCOV288807
INCOV288808
INCOV288809
INCOV288817
INCOV288818
INCOV288819
INCOV288820
INCOV288821
INCOV288822
INCOV288823
INCOV288824
INCOV288825
INCOV288826
INCOV288827
INCOV288828
INCOV288829
INCOV288830
INCOV288831
INCOV288832
INCOV289084
INCOV289627
INCOV290006
INCOV290007
INCOV290018
INCOV290035
INCOV290051
INCOV290194
INCOV288154
INCOV288226
INCOV288280
INCOV288281
INCOV288282
INCOV288314
INCOV288315
INCOV288316
INCOV288327
INCOV288332
INCOV288345
INCOV288354
INCOV288425
INCOV288426
INCOV288427
INCOV288465
INCOV288466
INCOV288484
INCOV288540
INCOV288614
INCOV288810
INCOV288811
INCOV289082
INCOV289577
INCOV289592
INCOV289602
INCOV289619
INCOV289654
INCOV290015
INCOV290016
INCOV290026
INCOV290036
INCOV290046
INCOV290052
INCOV290053
INCOV290072
INCOV290076
INCOV290100
INCOV291010
INCOV288153
INCOV288227
INCOV288243
INCOV288244
INCOV288263
INCOV288264
INCOV288265
INCOV288266
INCOV288277
INCOV288279
INCOV288317
INCOV288318
INCOV288365
INCOV288366
INCOV288392
INCOV288457
INCOV288541
INCOV288593
INCOV288813
INCOV288816
INCOV289062
INCOV289993
INCOV290075
INCOV290078
INCOV291011
INCOV292291
INCOV288274
INCOV288275
INCOV288276
INCOV288302
INCOV288303
INCOV288304
INCOV288305
INCOV288306
INCOV288307
INCOV288308
INCOV288328
INCOV288334
INCOV288338
INCOV288376
INCOV288428
INCOV288429
INCOV288430
INCOV288467
INCOV288542
INCOV288615
INCOV288616
INCOV288711
INCOV288712
INCOV289060
INCOV289061
INCOV289593
INCOV289621
INCOV289640
INCOV290028
INCOV290037
INCOV290047
INCOV290054
INCOV290073
INCOV290213
INCOV290963
INCOV292292
INCOV288273
INCOV288278
INCOV288309
INCOV288310
INCOV288311
INCOV288353
INCOV288377
INCOV288468
INCOV288620
INCOV288814
INCOV289580
INCOV289687
INCOV289695
INCOV289696
INCOV289704
INCOV290029
INCOV290030
INCOV290038
INCOV290055
INCOV290214
INCOV290964
INCOV292295
INCOV292296
INCOV288232
INCOV288233
INCOV288236
INCOV288351
INCOV288352
INCOV288367
INCOV288368
INCOV288369
INCOV288383
INCOV288417
INCOV288418
INCOV288419
INCOV288431
INCOV288432
INCOV288433
INCOV288434
INCOV288469
INCOV288470
INCOV288471
INCOV288472
INCOV288525
INCOV288526
INCOV288527
INCOV288528
INCOV288529
INCOV288543
INCOV288650
INCOV288689
INCOV288690
INCOV288782
INCOV288783
INCOV288784
INCOV288833
INCOV288834
INCOV288835
INCOV288836
INCOV288837
INCOV289058
INCOV289075
INCOV289499
INCOV289571
INCOV289583
INCOV289588
INCOV289589
INCOV289609
INCOV289618
INCOV289630
INCOV289641
INCOV289670
INCOV289718
INCOV289732
INCOV289735
INCOV289737
INCOV289756
INCOV289762
INCOV289769
INCOV290008
INCOV290014
INCOV290019
INCOV290041
INCOV290048
INCOV290056
INCOV290057
INCOV290058
INCOV290059
INCOV290079
INCOV290169
INCOV290170
INCOV290947
INCOV290965
INCOV290966
INCOV291012
INCOV291107
INCOV291108
INCOV291216
INCOV292249
INCOV292293
INCOV292294
INCOV292297
INCOV292298
INCOV288155
INCOV288230
INCOV288237
INCOV288249
INCOV288250
INCOV288370
INCOV288378
INCOV288379
INCOV288384
INCOV288458
INCOV288459
INCOV288516
INCOV288517
INCOV288518
INCOV288519
INCOV288520
INCOV288521
INCOV288522
INCOV288523
INCOV288524
INCOV288544
INCOV288545
INCOV288594
INCOV288632
INCOV288637
INCOV288642
INCOV288691
INCOV288692
INCOV288693
INCOV288694
INCOV288773
INCOV288774
INCOV288775
INCOV288777
INCOV288778
INCOV288785
INCOV288838
INCOV288839
INCOV289057
INCOV289066
INCOV289067
INCOV289086
INCOV289098
INCOV289128
INCOV289192
INCOV289193
INCOV289194
INCOV289207
INCOV289301
INCOV289302
INCOV289303
INCOV289304
INCOV289567
INCOV289568
INCOV289569
INCOV289570
INCOV289585
INCOV289586
INCOV289595
INCOV289605
INCOV289665
INCOV289666
INCOV289746
INCOV290020
INCOV290021
INCOV290022
INCOV290031
INCOV290060
INCOV290069
INCOV290080
INCOV290102
INCOV290940
INCOV290967
INCOV291109
INCOV291808
INCOV292299
INCOV293862
INCOV293871
INCOV293872
INCOV288371
INCOV288372
INCOV288373
INCOV288374
INCOV288375
INCOV288380
INCOV288381
INCOV288382
INCOV288385
INCOV288388
INCOV288393
INCOV288435
INCOV288436
INCOV288437
INCOV288438
INCOV288595
INCOV288596
INCOV288619
INCOV288633
INCOV288652
INCOV288776
INCOV288779
INCOV288780
INCOV288781
INCOV288786
INCOV288787
INCOV288788
INCOV288789
INCOV288790
INCOV288815
INCOV288862
INCOV288901
INCOV288902
INCOV289056
INCOV289071
INCOV289087
INCOV289099
INCOV289105
INCOV289107
INCOV289108
INCOV289109
INCOV289110
INCOV289111
INCOV289129
INCOV289139
INCOV289195
INCOV289197
INCOV289201
INCOV289202
INCOV289203
INCOV289204
INCOV289208
INCOV289235
INCOV289299
INCOV289500
INCOV289565
INCOV289566
INCOV289572
INCOV289573
INCOV289575
INCOV289578
INCOV289579
INCOV289582
INCOV289584
INCOV289594
INCOV289601
INCOV289610
INCOV289624
INCOV289645
INCOV289655
INCOV289657
INCOV289668
INCOV289692
INCOV289712
INCOV289715
INCOV289724
INCOV289738
INCOV289740
INCOV289744
INCOV289751
INCOV289764
INCOV289771
INCOV289994
INCOV289995
INCOV290032
INCOV290044
INCOV290061
INCOV290062
INCOV290063
INCOV290081
INCOV290082
INCOV290098
INCOV290153
INCOV290154
INCOV290171
INCOV290195
INCOV290215
INCOV290941
INCOV290948
INCOV290952
INCOV290953
INCOV290954
INCOV290955
INCOV290959
INCOV290968
INCOV290969
INCOV290976
INCOV290981
INCOV290982
INCOV290983
INCOV290984
INCOV290995
INCOV290998
INCOV291013
INCOV291014
INCOV291110
INCOV291111
INCOV291345
INCOV291346
INCOV292300
INCOV292301
INCOV292302
INCOV292303
INCOV292304
INCOV292305
INCOV288251
INCOV288252
INCOV288386
INCOV288387
INCOV288389
INCOV288391
INCOV288439
INCOV288440
INCOV288441
INCOV288442
INCOV288634
INCOV288643
INCOV288644
INCOV288645
INCOV288651
INCOV288653
INCOV288654
INCOV288655
INCOV288656
INCOV288657
INCOV288658
INCOV288659
INCOV288660
INCOV288661
INCOV288680
INCOV288695
INCOV288696
INCOV288713
INCOV288715
INCOV288721
INCOV288722
INCOV288726
INCOV288841
INCOV288842
INCOV288843
INCOV288852
INCOV288903
INCOV289063
INCOV289070
INCOV289088
INCOV289089
INCOV289090
INCOV289091
INCOV289092
INCOV289112
INCOV289130
INCOV289131
INCOV289132
INCOV289133
INCOV289138
INCOV289149
INCOV289150
INCOV289153
INCOV289154
INCOV289155
INCOV289156
INCOV289157
INCOV289185
INCOV289186
INCOV289234
INCOV289296
INCOV289297
INCOV289298
INCOV289501
INCOV289502
INCOV289503
INCOV289504
INCOV289505
INCOV289596
INCOV289598
INCOV289604
INCOV289606
INCOV289608
INCOV289614
INCOV289615
INCOV289617
INCOV289623
INCOV289632
INCOV289634
INCOV289636
INCOV289639
INCOV289644
INCOV289646
INCOV289650
INCOV289652
INCOV289658
INCOV289669
INCOV289674
INCOV289676
INCOV289677
INCOV289678
INCOV289679
INCOV289680
INCOV289683
INCOV289694
INCOV289698
INCOV289701
INCOV289705
INCOV289714
INCOV289719
INCOV289720
INCOV289721
INCOV289726
INCOV289733
INCOV289736
INCOV289741
INCOV289742
INCOV289743
INCOV289750
INCOV289752
INCOV289754
INCOV289755
INCOV289757
INCOV289759
INCOV289761
INCOV289763
INCOV289765
INCOV289766
INCOV289768
INCOV289770
INCOV289772
INCOV290005
INCOV290033
INCOV290039
INCOV290045
INCOV290064
INCOV290065
INCOV290066
INCOV290096
INCOV290158
INCOV290207
INCOV290942
INCOV290949
INCOV290956
INCOV290957
INCOV290960
INCOV290977
INCOV290996
INCOV290997
INCOV290999
INCOV291015
INCOV291112
INCOV291113
INCOV291185
INCOV291186
INCOV291837
INCOV291838
INCOV292078
INCOV292306
INCOV292307
INCOV292308
INCOV292309
INCOV292310
INCOV293840
INCOV293860
INCOV288443
INCOV288444
INCOV288445
INCOV288446
INCOV288447
INCOV288448
INCOV288449
INCOV288450
INCOV288662
INCOV288663
INCOV288664
INCOV288697
INCOV288698
INCOV288699
INCOV288700
INCOV288701
INCOV288707
INCOV288708
INCOV288719
INCOV288723
INCOV288727
INCOV288733
INCOV288840
INCOV288844
INCOV288845
INCOV288846
INCOV288847
INCOV288848
INCOV288849
INCOV288850
INCOV288851
INCOV289093
INCOV289094
INCOV289113
INCOV289114
INCOV289115
INCOV289116
INCOV289134
INCOV289140
INCOV289144
INCOV289145
INCOV289146
INCOV289151
INCOV289152
INCOV289158
INCOV289159
INCOV289160
INCOV289161
INCOV289163
INCOV289177
INCOV289189
INCOV289305
INCOV289506
INCOV289507
INCOV289508
INCOV289599
INCOV289603
INCOV289616
INCOV289625
INCOV289626
INCOV289628
INCOV289648
INCOV289660
INCOV289661
INCOV289671
INCOV289681
INCOV289685
INCOV289691
INCOV289702
INCOV289703
INCOV289708
INCOV289709
INCOV289710
INCOV289730
INCOV289749
INCOV289753
INCOV289758
INCOV289773
INCOV289774
INCOV289775
INCOV289996
INCOV289997
INCOV290023
INCOV290024
INCOV290027
INCOV290034
INCOV290042
INCOV290043
INCOV290067
INCOV290097
INCOV290110
INCOV290130
INCOV290131
INCOV290159
INCOV290216
INCOV290943
INCOV290958
INCOV290961
INCOV290978
INCOV290979
INCOV291000
INCOV291001
INCOV291002
INCOV291114
INCOV291349
INCOV291350
INCOV291351
INCOV291352
INCOV291353
INCOV291354
INCOV291355
INCOV291391
INCOV292248
INCOV292311
INCOV288253
INCOV288254
INCOV288451
INCOV288452
INCOV288453
INCOV288454
INCOV288455
INCOV288530
INCOV288531
INCOV288532
INCOV288621
INCOV288622
INCOV288623
INCOV288624
INCOV288666
INCOV288667
INCOV288668
INCOV288669
INCOV288702
INCOV288703
INCOV288704
INCOV288709
INCOV288716
INCOV288717
INCOV288718
INCOV288724
INCOV288728
INCOV288729
INCOV288730
INCOV288731
INCOV288734
INCOV288853
INCOV288870
INCOV288871
INCOV288872
INCOV288873
INCOV288874
INCOV289064
INCOV289072
INCOV289073
INCOV289074
INCOV289097
INCOV289106
INCOV289117
INCOV289118
INCOV289119
INCOV289120
INCOV289121
INCOV289122
INCOV289123
INCOV289124
INCOV289135
INCOV289136
INCOV289142
INCOV289143
INCOV289147
INCOV289165
INCOV289168
INCOV289169
INCOV289170
INCOV289171
INCOV289172
INCOV289173
INCOV289174
INCOV289175
INCOV289181
INCOV289182
INCOV289187
INCOV289188
INCOV289198
INCOV289209
INCOV289218
INCOV289219
INCOV289220
INCOV289221
INCOV289222
INCOV289509
INCOV289510
INCOV289515
INCOV289597
INCOV289600
INCOV289612
INCOV289620
INCOV289629
INCOV289643
INCOV289649
INCOV289651
INCOV289675
INCOV289684
INCOV289697
INCOV289706
INCOV289713
INCOV289716
INCOV289717
INCOV289723
INCOV289725
INCOV289728
INCOV289747
INCOV289803
INCOV289898
INCOV290002
INCOV290049
INCOV290068
INCOV290103
INCOV290135
INCOV290138
INCOV290141
INCOV290142
INCOV290172
INCOV290173
INCOV290206
INCOV290217
INCOV290218
INCOV290219
INCOV290944
INCOV290945
INCOV290970
INCOV291003
INCOV291004
INCOV291016
INCOV291115
INCOV291116
INCOV291117
INCOV291118
INCOV291119
INCOV291120
INCOV291121
INCOV291187
INCOV291188
INCOV291189
INCOV291190
INCOV291191
INCOV291192
INCOV291218
INCOV291365
INCOV291811
INCOV292079
INCOV288422
INCOV288670
INCOV288671
INCOV288672
INCOV288673
INCOV288674
INCOV288675
INCOV288676
INCOV288677
INCOV288678
INCOV288679
INCOV288705
INCOV288706
INCOV288732
INCOV288875
INCOV288876
INCOV288877
INCOV289096
INCOV289125
INCOV289141
INCOV289166
INCOV289183
INCOV289191
INCOV289210
INCOV289211
INCOV289212
INCOV289237
INCOV289238
INCOV289239
INCOV289240
INCOV289394
INCOV289427
INCOV289511
INCOV289512
INCOV289513
INCOV289514
INCOV289611
INCOV289633
INCOV289637
INCOV289647
INCOV289659
INCOV289662
INCOV289667
INCOV289672
INCOV289686
INCOV289689
INCOV289699
INCOV289707
INCOV289722
INCOV289729
INCOV290040
INCOV290050
INCOV290077
INCOV290101
INCOV290104
INCOV290109
INCOV290139
INCOV290146
INCOV290147
INCOV290148
INCOV290160
INCOV290220
INCOV291005
INCOV291006
INCOV291072
INCOV291075
INCOV291366
INCOV292312
INCOV292313
INCOV292314
INCOV292315
INCOV292316
INCOV293834
INCOV288625
INCOV288714
INCOV288720
INCOV288725
INCOV288746
INCOV288747
INCOV288748
INCOV288749
INCOV288758
INCOV288759
INCOV288761
INCOV288762
INCOV288769
INCOV288868
INCOV288880
INCOV288881
INCOV288882
INCOV289126
INCOV289148
INCOV289162
INCOV289164
INCOV289176
INCOV289178
INCOV289179
INCOV289180
INCOV289190
INCOV289213
INCOV289223
INCOV289225
INCOV289226
INCOV289292
INCOV289294
INCOV289295
INCOV289300
INCOV289306
INCOV289428
INCOV289429
INCOV289622
INCOV289635
INCOV289642
INCOV289653
INCOV289656
INCOV289673
INCOV289688
INCOV289693
INCOV289727
INCOV289734
INCOV289760
INCOV289796
INCOV290105
INCOV290116
INCOV290117
INCOV290118
INCOV290119
INCOV290121
INCOV290126
INCOV290161
INCOV290174
INCOV290175
INCOV290176
INCOV290177
INCOV290962
INCOV291073
INCOV291074
INCOV291122
INCOV291123
INCOV291124
INCOV291125
INCOV291126
INCOV291127
INCOV291128
INCOV291157
INCOV291193
INCOV291194
INCOV291356
INCOV291357
INCOV291369
INCOV291823
INCOV292244
INCOV292317
INCOV293888
INCOV293999
INCOV288636
INCOV288641
INCOV288646
INCOV288647
INCOV288648
INCOV288649
INCOV288665
INCOV288745
INCOV288750
INCOV288751
INCOV288757
INCOV288760
INCOV288763
INCOV288764
INCOV288854
INCOV288855
INCOV288856
INCOV288878
INCOV288879
INCOV288883
INCOV288884
INCOV288890
INCOV288891
INCOV288892
INCOV289065
INCOV289076
INCOV289100
INCOV289167
INCOV289184
INCOV289227
INCOV289241
INCOV289242
INCOV289243
INCOV289244
INCOV289307
INCOV289308
INCOV289310
INCOV289430
INCOV289431
INCOV289748
INCOV289777
INCOV289797
INCOV289800
INCOV289899
INCOV289900
INCOV289901
INCOV290111
INCOV290127
INCOV290129
INCOV290132
INCOV290143
INCOV290162
INCOV290178
INCOV290196
INCOV290221
INCOV290222
INCOV290223
INCOV291007
INCOV291008
INCOV291009
INCOV291017
INCOV291076
INCOV291129
INCOV291130
INCOV291131
INCOV291132
INCOV291133
INCOV291134
INCOV291158
INCOV291162
INCOV291163
INCOV291203
INCOV291204
INCOV291205
INCOV291314
INCOV291330
INCOV291358
INCOV291359
INCOV291360
INCOV291361
INCOV291362
INCOV291363
INCOV291364
INCOV291371
INCOV291372
INCOV291373
INCOV291374
INCOV291375
INCOV291377
INCOV291378
INCOV291420
INCOV291630
INCOV291824
INCOV292069
INCOV292318
INCOV293863
INCOV288461
INCOV288735
INCOV288736
INCOV288737
INCOV288738
INCOV288739
INCOV288740
INCOV288741
INCOV288742
INCOV288743
INCOV288744
INCOV288752
INCOV288753
INCOV288754
INCOV288755
INCOV288765
INCOV288857
INCOV288863
INCOV288885
INCOV288886
INCOV288887
INCOV288888
INCOV288889
INCOV289095
INCOV289101
INCOV289196
INCOV289205
INCOV289206
INCOV289214
INCOV289215
INCOV289228
INCOV289293
INCOV289309
INCOV289426
INCOV289471
INCOV289711
INCOV289801
INCOV289902
INCOV290000
INCOV290115
INCOV290120
INCOV290136
INCOV290179
INCOV290180
INCOV290197
INCOV290198
INCOV290199
INCOV290201
INCOV290208
INCOV290209
INCOV290224
INCOV290225
INCOV290985
INCOV290987
INCOV290988
INCOV290989
INCOV291032
INCOV291033
INCOV291034
INCOV291035
INCOV291036
INCOV291037
INCOV291038
INCOV291039
INCOV291040
INCOV291060
INCOV291061
INCOV291062
INCOV291063
INCOV291064
INCOV291065
INCOV291066
INCOV291077
INCOV291078
INCOV291079
INCOV291135
INCOV291136
INCOV291137
INCOV291138
INCOV291139
INCOV291140
INCOV291159
INCOV291160
INCOV291161
INCOV291164
INCOV291165
INCOV291166
INCOV291167
INCOV291195
INCOV291196
INCOV291197
INCOV291206
INCOV291217
INCOV291367
INCOV291368
INCOV291370
INCOV291379
INCOV291380
INCOV291381
INCOV291382
INCOV291383
INCOV291392
INCOV291396
INCOV291639
INCOV291648
INCOV291649
INCOV291650
INCOV291651
INCOV291652
INCOV291781
INCOV291880
INCOV292491
INCOV293889
INCOV293890
INCOV293891
INCOV293994
INCOV288551
INCOV288552
INCOV288553
INCOV288554
INCOV288555
INCOV288556
INCOV288557
INCOV288558
INCOV288559
INCOV288560
INCOV288561
INCOV288562
INCOV288563
INCOV288564
INCOV288567
INCOV288568
INCOV288756
INCOV288766
INCOV288767
INCOV288768
INCOV288770
INCOV288771
INCOV288865
INCOV289102
INCOV289103
INCOV289104
INCOV289216
INCOV289224
INCOV289229
INCOV289230
INCOV289231
INCOV289245
INCOV289246
INCOV289400
INCOV289410
INCOV289418
INCOV289419
INCOV289420
INCOV289421
INCOV289422
INCOV289423
INCOV289424
INCOV289425
INCOV289432
INCOV289433
INCOV289439
INCOV289440
INCOV289441
INCOV289472
INCOV289474
INCOV289516
INCOV289517
INCOV289518
INCOV289519
INCOV289520
INCOV289778
INCOV289781
INCOV289788
INCOV289791
INCOV289904
INCOV289905
INCOV289990
INCOV289998
INCOV290122
INCOV290137
INCOV290144
INCOV290155
INCOV290163
INCOV290164
INCOV290181
INCOV290182
INCOV290183
INCOV290184
INCOV290203
INCOV290210
INCOV290226
INCOV290951
INCOV290974
INCOV290975
INCOV290986
INCOV291041
INCOV291042
INCOV291043
INCOV291044
INCOV291045
INCOV291046
INCOV291067
INCOV291068
INCOV291080
INCOV291081
INCOV291082
INCOV291083
INCOV291084
INCOV291085
INCOV291086
INCOV291141
INCOV291142
INCOV291143
INCOV291144
INCOV291145
INCOV291146
INCOV291147
INCOV291148
INCOV291168
INCOV291169
INCOV291170
INCOV291171
INCOV291172
INCOV291173
INCOV291174
INCOV291175
INCOV291176
INCOV291177
INCOV291178
INCOV291221
INCOV291315
INCOV291319
INCOV291320
INCOV291321
INCOV291322
INCOV291323
INCOV291324
INCOV291325
INCOV291326
INCOV291327
INCOV291328
INCOV291329
INCOV291341
INCOV291347
INCOV291348
INCOV291376
INCOV291385
INCOV291386
INCOV291387
INCOV291393
INCOV291397
INCOV291427
INCOV291653
INCOV291654
INCOV291655
INCOV291656
INCOV291657
INCOV291658
INCOV291659
INCOV291757
INCOV291825
INCOV291826
INCOV291839
INCOV292252
INCOV292275
INCOV292319
INCOV293861
INCOV293874
INCOV293875
INCOV293892
INCOV293893
INCOV293896
INCOV293993
INCOV293995
INCOV294000
INCOV288546
INCOV288547
INCOV288548
INCOV288549
INCOV288550
INCOV288864
INCOV288866
INCOV288893
INCOV288894
INCOV288895
INCOV288896
INCOV289199
INCOV289232
INCOV289233
INCOV289247
INCOV289250
INCOV289251
INCOV289252
INCOV289263
INCOV289287
INCOV289288
INCOV289291
INCOV289395
INCOV289396
INCOV289399
INCOV289401
INCOV289402
INCOV289406
INCOV289407
INCOV289409
INCOV289411
INCOV289412
INCOV289416
INCOV289417
INCOV289434
INCOV289435
INCOV289436
INCOV289437
INCOV289438
INCOV289442
INCOV289443
INCOV289444
INCOV289445
INCOV289446
INCOV289473
INCOV289475
INCOV289476
INCOV289638
INCOV289779
INCOV289780
INCOV289783
INCOV289784
INCOV289785
INCOV289793
INCOV289794
INCOV289804
INCOV289805
INCOV289897
INCOV289991
INCOV289999
INCOV290123
INCOV290124
INCOV290149
INCOV290150
INCOV290165
INCOV290185
INCOV290186
INCOV290200
INCOV290211
INCOV290227
INCOV290228
INCOV290231
INCOV290232
INCOV290234
INCOV290235
INCOV290236
INCOV290237
INCOV290238
INCOV290950
INCOV290990
INCOV291047
INCOV291048
INCOV291049
INCOV291087
INCOV291088
INCOV291089
INCOV291090
INCOV291091
INCOV291092
INCOV291149
INCOV291150
INCOV291151
INCOV291156
INCOV291179
INCOV291180
INCOV291181
INCOV291182
INCOV291183
INCOV291184
INCOV291224
INCOV291225
INCOV291316
INCOV291318
INCOV291331
INCOV291332
INCOV291388
INCOV291389
INCOV291390
INCOV291394
INCOV291395
INCOV291405
INCOV291411
INCOV291605
INCOV291606
INCOV291607
INCOV291631
INCOV291632
INCOV291633
INCOV291635
INCOV291660
INCOV291661
INCOV291662
INCOV291663
INCOV291668
INCOV291758
INCOV291782
INCOV291783
INCOV291784
INCOV291785
INCOV291786
INCOV291787
INCOV291827
INCOV292070
INCOV292071
INCOV292267
INCOV292349
INCOV292492
INCOV292493
INCOV293858
INCOV293894
INCOV293895
INCOV293897
INCOV293898
INCOV293899
INCOV293900
INCOV293901
INCOV293992
INCOV295398
INCOV295405
INCOV288867
INCOV288897
INCOV288898
INCOV288899
INCOV288900
INCOV289200
INCOV289217
INCOV289236
INCOV289248
INCOV289253
INCOV289254
INCOV289255
INCOV289256
INCOV289257
INCOV289258
INCOV289265
INCOV289266
INCOV289286
INCOV289289
INCOV289397
INCOV289398
INCOV289403
INCOV289404
INCOV289405
INCOV289408
INCOV289413
INCOV289414
INCOV289415
INCOV289453
INCOV289454
INCOV289455
INCOV289542
INCOV289543
INCOV289558
INCOV289613
INCOV289782
INCOV289795
INCOV289798
INCOV289799
INCOV289806
INCOV289822
INCOV289823
INCOV289824
INCOV289825
INCOV289826
INCOV289827
INCOV289828
INCOV289829
INCOV289830
INCOV289846
INCOV289847
INCOV289848
INCOV289849
INCOV289850
INCOV289851
INCOV289852
INCOV289853
INCOV290004
INCOV290125
INCOV290133
INCOV290134
INCOV290140
INCOV290151
INCOV290166
INCOV290167
INCOV290187
INCOV290188
INCOV290189
INCOV290204
INCOV290229
INCOV290939
INCOV290971
INCOV290991
INCOV290992
INCOV291025
INCOV291026
INCOV291050
INCOV291051
INCOV291093
INCOV291094
INCOV291095
INCOV291096
INCOV291097
INCOV291098
INCOV291099
INCOV291100
INCOV291101
INCOV291102
INCOV291103
INCOV291104
INCOV291105
INCOV291152
INCOV291153
INCOV291154
INCOV291155
INCOV291222
INCOV291317
INCOV291333
INCOV291342
INCOV291398
INCOV291399
INCOV291634
INCOV291636
INCOV291664
INCOV291665
INCOV291666
INCOV291667
INCOV291754
INCOV291755
INCOV291756
INCOV291759
INCOV291761
INCOV291762
INCOV291829
INCOV291831
INCOV291832
INCOV291855
INCOV291856
INCOV291889
INCOV291890
INCOV292157
INCOV292158
INCOV292159
INCOV292264
INCOV292266
INCOV292268
INCOV292269
INCOV292270
INCOV292321
INCOV292447
INCOV292448
INCOV292470
INCOV292516
INCOV293876
INCOV293877
INCOV293878
INCOV294001
INCOV294416
INCOV295438
INCOV288869
INCOV289249
INCOV289259
INCOV289264
INCOV289447
INCOV289448
INCOV289449
INCOV289450
INCOV289451
INCOV289452
INCOV289456
INCOV289457
INCOV289458
INCOV289459
INCOV289460
INCOV289461
INCOV289462
INCOV289463
INCOV289464
INCOV289465
INCOV289466
INCOV289467
INCOV289468
INCOV289469
INCOV289470
INCOV289731
INCOV289802
INCOV289807
INCOV289808
INCOV289809
INCOV289810
INCOV289811
INCOV290156
INCOV290168
INCOV290190
INCOV290230
INCOV290993
INCOV291069
INCOV291070
INCOV291106
INCOV291220
INCOV291422
INCOV291788
INCOV291789
INCOV291794
INCOV291795
INCOV291833
INCOV291974
INCOV292150
INCOV292271
INCOV292323
INCOV292449
INCOV289260
INCOV289261
INCOV289262
INCOV289267
INCOV289268
INCOV289269
INCOV289270
INCOV289271
INCOV289272
INCOV289273
INCOV289274
INCOV289275
INCOV289276
INCOV289277
INCOV289278
INCOV289521
INCOV289522
INCOV289540
INCOV289541
INCOV289557
INCOV289682
INCOV289789
INCOV289812
INCOV289813
INCOV289814
INCOV290003
INCOV290145
INCOV290152
INCOV290157
INCOV290191
INCOV290192
INCOV290193
INCOV290212
INCOV291018
INCOV291019
INCOV291020
INCOV291021
INCOV291022
INCOV291023
INCOV291024
INCOV291052
INCOV291053
INCOV291054
INCOV291055
INCOV291198
INCOV291223
INCOV291226
INCOV291313
INCOV291343
INCOV291401
INCOV291402
INCOV291403
INCOV291406
INCOV291642
INCOV291677
INCOV291678
INCOV291679
INCOV291680
INCOV291681
INCOV291682
INCOV291708
INCOV291710
INCOV291711
INCOV291712
INCOV291713
INCOV291714
INCOV291715
INCOV291716
INCOV291760
INCOV291763
INCOV291764
INCOV291765
INCOV291770
INCOV291796
INCOV291797
INCOV291798
INCOV291840
INCOV291857
INCOV291868
INCOV291891
INCOV291920
INCOV291952
INCOV292008
INCOV292009
INCOV292010
INCOV292117
INCOV292272
INCOV292273
INCOV292274
INCOV292285
INCOV292286
INCOV292287
INCOV292382
INCOV292383
INCOV292384
INCOV292385
INCOV292386
INCOV292387
INCOV292388
INCOV292389
INCOV292390
INCOV292391
INCOV292392
INCOV292393
INCOV292450
INCOV292471
INCOV292528
INCOV292560
INCOV292561
INCOV292562
INCOV293996
INCOV295408
INCOV289280
INCOV289290
INCOV289523
INCOV289786
INCOV289787
INCOV289790
INCOV289815
INCOV289816
INCOV289817
INCOV289818
INCOV289819
INCOV289820
INCOV289821
INCOV289831
INCOV289832
INCOV289833
INCOV289834
INCOV289835
INCOV289836
INCOV289837
INCOV290001
INCOV290205
INCOV290233
INCOV290239
INCOV290240
INCOV290972
INCOV290980
INCOV291056
INCOV291057
INCOV291058
INCOV291059
INCOV291199
INCOV291200
INCOV291207
INCOV291344
INCOV291400
INCOV291404
INCOV291407
INCOV291408
INCOV291410
INCOV291428
INCOV291438
INCOV291637
INCOV291641
INCOV291717
INCOV291718
INCOV291719
INCOV291766
INCOV291767
INCOV291768
INCOV291769
INCOV291776
INCOV291778
INCOV291779
INCOV291790
INCOV291791
INCOV291792
INCOV291820
INCOV291821
INCOV291843
INCOV291858
INCOV291869
INCOV291870
INCOV291882
INCOV291942
INCOV291943
INCOV291956
INCOV292118
INCOV292154
INCOV292155
INCOV292242
INCOV292322
INCOV292343
INCOV292394
INCOV292395
INCOV292396
INCOV292397
INCOV292398
INCOV292399
INCOV292400
INCOV292401
INCOV292402
INCOV292451
INCOV292452
INCOV292517
INCOV293859
INCOV293864
INCOV293879
INCOV293997
INCOV294086
INCOV294159
INCOV294160
INCOV294464
INCOV294465
INCOV294466
INCOV294538
INCOV295130
INCOV295414
INCOV295439
INCOV295440
INCOV289279
INCOV289281
INCOV289282
INCOV289283
INCOV289284
INCOV289285
INCOV289838
INCOV289839
INCOV289840
INCOV289841
INCOV289854
INCOV289855
INCOV289856
INCOV289857
INCOV289858
INCOV289859
INCOV289860
INCOV289893
INCOV289894
INCOV289903
INCOV290009
INCOV290010
INCOV290011
INCOV290973
INCOV291027
INCOV291028
INCOV291029
INCOV291201
INCOV291212
INCOV291213
INCOV291214
INCOV291215
INCOV291219
INCOV291334
INCOV291409
INCOV291413
INCOV291424
INCOV291439
INCOV291638
INCOV291669
INCOV291670
INCOV291720
INCOV291721
INCOV291722
INCOV291723
INCOV291724
INCOV291740
INCOV291771
INCOV291772
INCOV291773
INCOV291774
INCOV291775
INCOV291777
INCOV291780
INCOV291816
INCOV291867
INCOV291871
INCOV291892
INCOV291893
INCOV291921
INCOV291944
INCOV291945
INCOV291979
INCOV291980
INCOV291981
INCOV291982
INCOV291983
INCOV292067
INCOV292072
INCOV292080
INCOV292119
INCOV292240
INCOV292241
INCOV292247
INCOV292331
INCOV292404
INCOV292405
INCOV292406
INCOV292453
INCOV292454
INCOV292455
INCOV292529
INCOV292563
INCOV293835
INCOV293851
INCOV293867
INCOV293868
INCOV293869
INCOV294537
INCOV294546
INCOV294547
INCOV294548
INCOV294549
INCOV295134
INCOV295419
INCOV295496
INCOV289524
INCOV289525
INCOV289526
INCOV289527
INCOV289528
INCOV289529
INCOV289530
INCOV289531
INCOV289532
INCOV289533
INCOV289536
INCOV289538
INCOV289539
INCOV289842
INCOV289843
INCOV289844
INCOV289882
INCOV289883
INCOV289884
INCOV289885
INCOV289886
INCOV289887
INCOV289888
INCOV289889
INCOV289890
INCOV289891
INCOV290012
INCOV290013
INCOV291030
INCOV291031
INCOV291208
INCOV291209
INCOV291210
INCOV291211
INCOV291412
INCOV291414
INCOV291415
INCOV291416
INCOV291417
INCOV291430
INCOV291538
INCOV291539
INCOV291540
INCOV291541
INCOV291542
INCOV291543
INCOV291544
INCOV291545
INCOV291546
INCOV291547
INCOV291556
INCOV291557
INCOV291558
INCOV291559
INCOV291593
INCOV291594
INCOV291595
INCOV291596
INCOV291643
INCOV291644
INCOV291645
INCOV291646
INCOV291671
INCOV291672
INCOV291673
INCOV291725
INCOV291726
INCOV291727
INCOV291793
INCOV291813
INCOV291828
INCOV291841
INCOV291844
INCOV291864
INCOV291873
INCOV291876
INCOV291883
INCOV291894
INCOV291922
INCOV291935
INCOV291946
INCOV291953
INCOV291957
INCOV291958
INCOV291984
INCOV291985
INCOV292011
INCOV292012
INCOV292036
INCOV292056
INCOV292057
INCOV292238
INCOV292239
INCOV292288
INCOV292289
INCOV292290
INCOV292332
INCOV292350
INCOV292407
INCOV292408
INCOV292409
INCOV292410
INCOV292411
INCOV292456
INCOV292457
INCOV292458
INCOV292459
INCOV292460
INCOV292486
INCOV292518
INCOV292530
INCOV293865
INCOV293866
INCOV294375
INCOV294539
INCOV294540
INCOV294541
INCOV294542
INCOV294543
INCOV295137
INCOV295138
INCOV295139
INCOV295140
INCOV295141
INCOV295409
INCOV289845
INCOV289992
INCOV291335
INCOV291418
INCOV291419
INCOV291421
INCOV291423
INCOV291535
INCOV291536
INCOV291537
INCOV291548
INCOV291549
INCOV291561
INCOV291562
INCOV291563
INCOV291564
INCOV291565
INCOV291566
INCOV291567
INCOV291568
INCOV291569
INCOV291577
INCOV291578
INCOV291579
INCOV291580
INCOV291581
INCOV291597
INCOV291598
INCOV291647
INCOV291674
INCOV291675
INCOV291676
INCOV291701
INCOV291702
INCOV291703
INCOV291704
INCOV291705
INCOV291706
INCOV291728
INCOV291729
INCOV291730
INCOV291731
INCOV291732
INCOV291733
INCOV291734
INCOV291735
INCOV291736
INCOV291737
INCOV291738
INCOV291739
INCOV291741
INCOV291742
INCOV291743
INCOV291818
INCOV291830
INCOV291834
INCOV291842
INCOV291851
INCOV291854
INCOV291859
INCOV291860
INCOV291865
INCOV291874
INCOV291878
INCOV291879
INCOV291884
INCOV291923
INCOV291924
INCOV291936
INCOV291937
INCOV291947
INCOV291959
INCOV291960
INCOV291986
INCOV291987
INCOV291988
INCOV292013
INCOV292014
INCOV292015
INCOV292016
INCOV292017
INCOV292018
INCOV292019
INCOV292020
INCOV292065
INCOV292073
INCOV292075
INCOV292081
INCOV292082
INCOV292243
INCOV292333
INCOV292334
INCOV292335
INCOV292336
INCOV292337
INCOV292338
INCOV292353
INCOV292354
INCOV292412
INCOV292413
INCOV292414
INCOV292415
INCOV292416
INCOV292461
INCOV292462
INCOV292463
INCOV292519
INCOV292520
INCOV292564
INCOV292565
INCOV292566
INCOV293836
INCOV293848
INCOV293870
INCOV293940
INCOV293941
INCOV294374
INCOV294544
INCOV294545
INCOV294550
INCOV295143
INCOV295144
INCOV295147
INCOV295148
INCOV295399
INCOV295410
INCOV295411
INCOV289861
INCOV289862
INCOV289863
INCOV289864
INCOV289865
INCOV289866
INCOV289867
INCOV289868
INCOV289869
INCOV289870
INCOV289871
INCOV289872
INCOV289873
INCOV289874
INCOV289875
INCOV289876
INCOV289877
INCOV289878
INCOV289879
INCOV289880
INCOV289881
INCOV289892
INCOV290994
INCOV291425
INCOV291426
INCOV291433
INCOV291434
INCOV291550
INCOV291551
INCOV291553
INCOV291554
INCOV291555
INCOV291560
INCOV291570
INCOV291571
INCOV291582
INCOV291583
INCOV291584
INCOV291585
INCOV291586
INCOV291587
INCOV291588
INCOV291608
INCOV291640
INCOV291695
INCOV291696
INCOV291697
INCOV291698
INCOV291819
INCOV291835
INCOV291845
INCOV291861
INCOV291881
INCOV291885
INCOV291925
INCOV291926
INCOV291948
INCOV291961
INCOV291989
INCOV291990
INCOV291991
INCOV291992
INCOV292021
INCOV292022
INCOV292023
INCOV292024
INCOV292066
INCOV292074
INCOV292083
INCOV292099
INCOV292100
INCOV292120
INCOV292122
INCOV292123
INCOV292124
INCOV292148
INCOV292277
INCOV292278
INCOV292320
INCOV292339
INCOV292340
INCOV292417
INCOV292418
INCOV292419
INCOV292420
INCOV292421
INCOV292422
INCOV292423
INCOV292521
INCOV292522
INCOV292531
INCOV292567
INCOV293849
INCOV293850
INCOV293852
INCOV293904
INCOV293942
INCOV294083
INCOV294188
INCOV294370
INCOV295424
INCOV295427
INCOV295428
INCOV291436
INCOV291552
INCOV291599
INCOV291836
INCOV291852
INCOV291872
INCOV291875
INCOV291877
INCOV291886
INCOV291899
INCOV291917
INCOV291927
INCOV291928
INCOV291949
INCOV291962
INCOV291993
INCOV292149
INCOV292151
INCOV292276
INCOV292279
INCOV292280
INCOV292281
INCOV292494
INCOV292502
INCOV293880
INCOV294189
INCOV289544
INCOV289545
INCOV291429
INCOV291431
INCOV291432
INCOV291435
INCOV291437
INCOV291572
INCOV291573
INCOV291574
INCOV291575
INCOV291576
INCOV291589
INCOV291590
INCOV291591
INCOV291592
INCOV291600
INCOV291601
INCOV291602
INCOV291603
INCOV291604
INCOV291610
INCOV291622
INCOV291801
INCOV291814
INCOV291846
INCOV291847
INCOV291848
INCOV291862
INCOV291863
INCOV291866
INCOV291888
INCOV291895
INCOV291896
INCOV291900
INCOV291901
INCOV291902
INCOV291903
INCOV291904
INCOV291908
INCOV291938
INCOV291963
INCOV291975
INCOV291994
INCOV291995
INCOV291996
INCOV292037
INCOV292038
INCOV292039
INCOV292058
INCOV292059
INCOV292060
INCOV292061
INCOV292062
INCOV292063
INCOV292064
INCOV292076
INCOV292216
INCOV292217
INCOV292218
INCOV292219
INCOV292220
INCOV292245
INCOV292246
INCOV292284
INCOV292341
INCOV292344
INCOV292345
INCOV292355
INCOV292356
INCOV292357
INCOV292358
INCOV292424
INCOV292425
INCOV292426
INCOV292427
INCOV292428
INCOV292429
INCOV292430
INCOV292464
INCOV292465
INCOV292466
INCOV292467
INCOV292495
INCOV292496
INCOV292498
INCOV292503
INCOV292532
INCOV292533
INCOV292568
INCOV292569
INCOV292606
INCOV293881
INCOV294082
INCOV294085
INCOV294088
INCOV294141
INCOV294190
INCOV295152
INCOV291609
INCOV291611
INCOV291612
INCOV291614
INCOV291623
INCOV291624
INCOV291628
INCOV291629
INCOV291683
INCOV291684
INCOV291685
INCOV291686
INCOV291687
INCOV291688
INCOV291689
INCOV291691
INCOV291692
INCOV291694
INCOV291699
INCOV291707
INCOV291709
INCOV291744
INCOV291752
INCOV291753
INCOV291802
INCOV291849
INCOV291850
INCOV291853
INCOV291887
INCOV291897
INCOV291905
INCOV291906
INCOV291907
INCOV291910
INCOV291929
INCOV291930
INCOV291931
INCOV291939
INCOV291964
INCOV291976
INCOV292025
INCOV292026
INCOV292034
INCOV292040
INCOV292068
INCOV292077
INCOV292098
INCOV292125
INCOV292126
INCOV292135
INCOV292136
INCOV292221
INCOV292222
INCOV292223
INCOV292228
INCOV292359
INCOV292360
INCOV292361
INCOV292431
INCOV292432
INCOV292433
INCOV292434
INCOV292435
INCOV292468
INCOV292499
INCOV292523
INCOV292570
INCOV292571
INCOV292607
INCOV292608
INCOV292609
INCOV292610
INCOV292612
INCOV292613
INCOV292649
INCOV292655
INCOV292656
INCOV292657
INCOV293841
INCOV293847
INCOV293882
INCOV293998
INCOV294078
INCOV294079
INCOV294081
INCOV294090
INCOV294191
INCOV294282
INCOV294283
INCOV294371
INCOV294551
INCOV294638
INCOV295415
INCOV291613
INCOV291615
INCOV291616
INCOV291617
INCOV291618
INCOV291619
INCOV291620
INCOV291625
INCOV291803
INCOV291898
INCOV291911
INCOV291912
INCOV291918
INCOV291932
INCOV291933
INCOV291940
INCOV291965
INCOV291966
INCOV291997
INCOV291998
INCOV292027
INCOV292028
INCOV292029
INCOV292041
INCOV292042
INCOV292043
INCOV292121
INCOV292127
INCOV292143
INCOV292144
INCOV292145
INCOV292146
INCOV292147
INCOV292152
INCOV292153
INCOV292156
INCOV292282
INCOV292283
INCOV292362
INCOV292363
INCOV292364
INCOV292365
INCOV292366
INCOV292367
INCOV292368
INCOV292369
INCOV292436
INCOV292437
INCOV292438
INCOV292439
INCOV292469
INCOV292500
INCOV292501
INCOV292504
INCOV292505
INCOV292506
INCOV292509
INCOV292524
INCOV292534
INCOV292572
INCOV292602
INCOV292641
INCOV292642
INCOV292643
INCOV292644
INCOV292650
INCOV292651
INCOV293846
INCOV293883
INCOV293884
INCOV293969
INCOV293970
INCOV294080
INCOV294084
INCOV294087
INCOV294089
INCOV294368
INCOV294552
INCOV295154
INCOV295156
INCOV295429
INCOV295486
INCOV295487
INCOV291626
INCOV291690
INCOV291693
INCOV291700
INCOV291745
INCOV291746
INCOV291804
INCOV291815
INCOV291913
INCOV291914
INCOV291915
INCOV291934
INCOV291950
INCOV291954
INCOV291955
INCOV291967
INCOV291968
INCOV291972
INCOV291973
INCOV291977
INCOV291978
INCOV291999
INCOV292000
INCOV292001
INCOV292002
INCOV292030
INCOV292031
INCOV292044
INCOV292045
INCOV292046
INCOV292047
INCOV292048
INCOV292049
INCOV292101
INCOV292102
INCOV292103
INCOV292104
INCOV292105
INCOV292106
INCOV292107
INCOV292342
INCOV292370
INCOV292371
INCOV292372
INCOV292440
INCOV292508
INCOV292510
INCOV292525
INCOV292536
INCOV292537
INCOV292542
INCOV292543
INCOV292573
INCOV292603
INCOV292614
INCOV292615
INCOV292616
INCOV292617
INCOV292645
INCOV292658
INCOV293873
INCOV293885
INCOV294091
INCOV294092
INCOV294094
INCOV294112
INCOV294192
INCOV294679
INCOV294682
INCOV294683
INCOV294684
INCOV294954
INCOV294976
INCOV291747
INCOV291748
INCOV291805
INCOV291909
INCOV291916
INCOV291941
INCOV291951
INCOV291969
INCOV291970
INCOV291971
INCOV292003
INCOV292004
INCOV292005
INCOV292032
INCOV292033
INCOV292050
INCOV292051
INCOV292108
INCOV292109
INCOV292110
INCOV292111
INCOV292112
INCOV292113
INCOV292351
INCOV292373
INCOV292374
INCOV292375
INCOV292376
INCOV292377
INCOV292378
INCOV292441
INCOV292442
INCOV292443
INCOV292444
INCOV292445
INCOV292480
INCOV292497
INCOV292507
INCOV292511
INCOV292514
INCOV292538
INCOV292544
INCOV292546
INCOV292574
INCOV292575
INCOV292579
INCOV292604
INCOV292611
INCOV292618
INCOV292619
INCOV292620
INCOV292640
INCOV292646
INCOV293842
INCOV293902
INCOV293923
INCOV294138
INCOV294142
INCOV294157
INCOV294161
INCOV294193
INCOV294208
INCOV294209
INCOV294210
INCOV294211
INCOV294212
INCOV294213
INCOV294284
INCOV294369
INCOV294440
INCOV294680
INCOV294685
INCOV295400
INCOV291621
INCOV291749
INCOV291750
INCOV291751
INCOV291806
INCOV292006
INCOV292007
INCOV292052
INCOV292053
INCOV292054
INCOV292346
INCOV292347
INCOV292352
INCOV292379
INCOV292380
INCOV292381
INCOV292446
INCOV292477
INCOV292512
INCOV292526
INCOV292527
INCOV292547
INCOV292548
INCOV292549
INCOV292550
INCOV292551
INCOV292552
INCOV292576
INCOV292577
INCOV292647
INCOV292648
INCOV293886
INCOV293905
INCOV293971
INCOV293977
INCOV294093
INCOV294140
INCOV294194
INCOV294367
INCOV294417
INCOV294418
INCOV294681
INCOV294977
INCOV294978
INCOV295488
INCOV295489
INCOV295787
INCOV291627
INCOV291919
INCOV292114
INCOV292115
INCOV292116
INCOV292128
INCOV292129
INCOV292130
INCOV292131
INCOV292132
INCOV292133
INCOV292134
INCOV292137
INCOV292138
INCOV292139
INCOV292140
INCOV292141
INCOV292545
INCOV292553
INCOV292596
INCOV292652
INCOV292653
INCOV292654
INCOV293843
INCOV293906
INCOV294118
INCOV294139
INCOV294285
INCOV294686
INCOV292035
INCOV292055
INCOV292226
INCOV292227
INCOV292231
INCOV292348
INCOV292475
INCOV292476
INCOV292481
INCOV292482
INCOV292485
INCOV292535
INCOV292539
INCOV292540
INCOV292541
INCOV292554
INCOV292555
INCOV292556
INCOV292557
INCOV292578
INCOV292589
INCOV292590
INCOV292621
INCOV292622
INCOV292623
INCOV292624
INCOV293837
INCOV293912
INCOV293913
INCOV293914
INCOV293924
INCOV293972
INCOV294134
INCOV294366
INCOV294647
INCOV294648
INCOV294979
INCOV291822
INCOV292142
INCOV292232
INCOV292478
INCOV292479
INCOV292513
INCOV292558
INCOV292580
INCOV292581
INCOV292591
INCOV292592
INCOV292625
INCOV292662
INCOV293831
INCOV293832
INCOV293887
INCOV293915
INCOV293920
INCOV293921
INCOV293922
INCOV293939
INCOV294095
INCOV294115
INCOV294137
INCOV294214
INCOV294215
INCOV294286
INCOV294365
INCOV294409
INCOV294410
INCOV294419
INCOV294420
INCOV294441
INCOV294649
INCOV294650
INCOV294651
INCOV294687
INCOV294688
INCOV294980
INCOV295018
INCOV295019
INCOV295158
INCOV295490
INCOV295491
INCOV292224
INCOV292229
INCOV292230
INCOV292472
INCOV292483
INCOV292484
INCOV292559
INCOV292582
INCOV292583
INCOV292584
INCOV292585
INCOV292586
INCOV292587
INCOV292593
INCOV292626
INCOV292627
INCOV292628
INCOV292629
INCOV292630
INCOV292659
INCOV293833
INCOV293838
INCOV293839
INCOV293916
INCOV293917
INCOV293918
INCOV293919
INCOV294103
INCOV294110
INCOV294111
INCOV294216
INCOV294253
INCOV294411
INCOV294412
INCOV294421
INCOV294652
INCOV295492
INCOV295493
INCOV292588
INCOV292594
INCOV292605
INCOV292631
INCOV292660
INCOV293933
INCOV293973
INCOV294135
INCOV294205
INCOV294414
INCOV294422
INCOV294423
INCOV294428
INCOV294653
INCOV294654
INCOV294655
INCOV294689
INCOV294981
INCOV292225
INCOV292595
INCOV292632
INCOV292633
INCOV292634
INCOV292635
INCOV292636
INCOV292637
INCOV293935
INCOV293936
INCOV293962
INCOV293985
INCOV294125
INCOV294126
INCOV294127
INCOV294128
INCOV294206
INCOV294278
INCOV294363
INCOV294364
INCOV294372
INCOV294424
INCOV294425
INCOV294426
INCOV294427
INCOV294632
INCOV294656
INCOV294657
INCOV294658
INCOV294659
INCOV294660
INCOV294661
INCOV294690
INCOV294982
INCOV295032
INCOV295033
INCOV295494
INCOV292515
INCOV292597
INCOV292598
INCOV292601
INCOV292661
INCOV292664
INCOV293844
INCOV293845
INCOV293925
INCOV293926
INCOV293927
INCOV293928
INCOV293929
INCOV293930
INCOV293931
INCOV293934
INCOV293946
INCOV293961
INCOV293963
INCOV293974
INCOV294129
INCOV294136
INCOV294172
INCOV294173
INCOV294174
INCOV294175
INCOV294195
INCOV294207
INCOV294413
INCOV294429
INCOV294442
INCOV294662
INCOV294663
INCOV294664
INCOV294665
INCOV294666
INCOV295034
INCOV295035
INCOV295036
INCOV295037
INCOV295788
INCOV295789
INCOV295790
INCOV295791
INCOV292473
INCOV292474
INCOV293975
INCOV294130
INCOV294176
INCOV294434
INCOV292599
INCOV292600
INCOV292638
INCOV293957
INCOV293976
INCOV294114
INCOV294373
INCOV294415
INCOV294443
INCOV294444
INCOV294445
INCOV294667
INCOV294668
INCOV294669
INCOV292639
INCOV293932
INCOV293964
INCOV293965
INCOV294352
INCOV294353
INCOV294633
INCOV294670
INCOV294968
INCOV295038
INCOV295112
INCOV292663
INCOV293830
INCOV293943
INCOV293944
INCOV293945
INCOV293978
INCOV293980
INCOV294113
INCOV294131
INCOV294132
INCOV294133
INCOV294196
INCOV294430
INCOV294431
INCOV294435
INCOV294449
INCOV294631
INCOV294671
INCOV294672
INCOV294790
INCOV294877
INCOV295056
INCOV295057
INCOV295058
INCOV295059
INCOV295060
INCOV295061
INCOV295063
INCOV295792
INCOV293947
INCOV293948
INCOV294155
INCOV294156
INCOV294158
INCOV294162
INCOV294217
INCOV294218
INCOV294219
INCOV294220
INCOV294221
INCOV294222
INCOV294223
INCOV294432
INCOV294433
INCOV294436
INCOV294450
INCOV294983
INCOV295062
INCOV295064
INCOV295065
INCOV295089
INCOV295090
INCOV295159
INCOV295160
INCOV293979
INCOV294107
INCOV294438
INCOV295091
INCOV293949
INCOV293966
INCOV294124
INCOV294354
INCOV294355
INCOV294437
INCOV294446
INCOV294447
INCOV294448
INCOV294451
INCOV294452
INCOV294635
INCOV294673
INCOV294674
INCOV294988
INCOV295066
INCOV295067
INCOV295068
INCOV295069
INCOV295853
INCOV293981
INCOV293982
INCOV293984
INCOV294119
INCOV293983
INCOV294106
INCOV294109
INCOV294120
INCOV294121
INCOV294123
INCOV294143
INCOV294144
INCOV294145
INCOV294146
INCOV294165
INCOV294254
INCOV294255
INCOV294256
INCOV294257
INCOV294357
INCOV294358
INCOV294439
INCOV294453
INCOV294454
INCOV294455
INCOV294456
INCOV294458
INCOV294459
INCOV294460
INCOV294476
INCOV294477
INCOV294506
INCOV294634
INCOV294636
INCOV294640
INCOV294675
INCOV294708
INCOV294984
INCOV295039
INCOV295040
INCOV295041
INCOV295042
INCOV295043
INCOV295044
INCOV295045
INCOV295070
INCOV293950
INCOV293951
INCOV293952
INCOV293953
INCOV293954
INCOV293958
INCOV294104
INCOV294105
INCOV294166
INCOV294199
INCOV294258
INCOV294359
INCOV294376
INCOV294467
INCOV294468
INCOV294469
INCOV294637
INCOV294709
INCOV294985
INCOV295071
INCOV295072
INCOV295073
INCOV295074
INCOV295075
INCOV295076
INCOV295077
INCOV295793
INCOV295854
INCOV295855
INCOV295856
INCOV293955
INCOV293956
INCOV293967
INCOV294108
INCOV294116
INCOV294122
INCOV294200
INCOV294224
INCOV294457
INCOV294461
INCOV294462
INCOV294463
INCOV294470
INCOV294471
INCOV294478
INCOV294479
INCOV294480
INCOV294485
INCOV294486
INCOV294487
INCOV294641
INCOV294642
INCOV294676
INCOV295078
INCOV295857
INCOV293937
INCOV293938
INCOV294077
INCOV294102
INCOV294197
INCOV294259
INCOV294295
INCOV294350
INCOV294351
INCOV294356
INCOV294379
INCOV294472
INCOV294473
INCOV294481
INCOV294643
INCOV294644
INCOV295012
INCOV295079
INCOV295080
INCOV295081
INCOV295858
INCOV295859
INCOV295860
INCOV295861
INCOV294275
INCOV294735
INCOV294741
INCOV294742
INCOV294745
INCOV294898
INCOV294899
INCOV294900
INCOV295233
INCOV295234
INCOV295235
INCOV295236
INCOV295237
INCOV295238
INCOV295248
INCOV295249
INCOV295497
INCOV295869
INCOV295947
INCOV295319
INCOV295320
INCOV295321
INCOV295322
INCOV295449
INCOV295635
INCOV295636
INCOV295637
INCOV295651
INCOV295769
INCOV295917
INCOV295951
INCOV295975
INCOV295999
INCOV295990
INCOV294075
INCOV294076
INCOV294096
INCOV294287
INCOV294288
INCOV294289
INCOV294290
INCOV294291
INCOV294296
INCOV294475
INCOV294482
INCOV294483
INCOV294553
INCOV294554
INCOV294677
INCOV294678
INCOV295016
INCOV295086
INCOV295161
INCOV294706
INCOV294737
INCOV294738
INCOV294739
INCOV294743
INCOV294746
INCOV294748
INCOV294756
INCOV294821
INCOV294853
INCOV294854
INCOV294855
INCOV294901
INCOV294902
INCOV294990
INCOV294991
INCOV295229
INCOV295230
INCOV295239
INCOV295240
INCOV295250
INCOV295251
INCOV295252
INCOV295673
INCOV295674
INCOV295678
INCOV295679
INCOV295683
INCOV295684
INCOV295716
INCOV295316
INCOV295317
INCOV295318
INCOV295450
INCOV295613
INCOV295614
INCOV295615
INCOV295616
INCOV295617
INCOV295618
INCOV295619
INCOV295620
INCOV295621
INCOV295638
INCOV295639
INCOV295652
INCOV295653
INCOV295777
INCOV295890
INCOV295918
INCOV295919
INCOV295889
INCOV295906
INCOV295909
INCOV295943
INCOV293959
INCOV293968
INCOV294097
INCOV294099
INCOV294100
INCOV294101
INCOV294198
INCOV294377
INCOV294381
INCOV294382
INCOV294402
INCOV294484
INCOV294488
INCOV294489
INCOV294555
INCOV294556
INCOV294557
INCOV294588
INCOV294589
INCOV294639
INCOV294646
INCOV295082
INCOV295083
INCOV295084
INCOV295085
INCOV295227
INCOV295862
INCOV295863
INCOV294740
INCOV295672
INCOV295680
INCOV295681
INCOV295682
INCOV295313
INCOV295314
INCOV295315
INCOV295451
INCOV295640
INCOV295775
INCOV295776
INCOV295800
INCOV295891
INCOV295920
INCOV295896
INCOV296015
INCOV296016
INCOV295991
INCOV295992
INCOV293960
INCOV294098
INCOV294378
INCOV294590
INCOV294707
INCOV294744
INCOV294749
INCOV294757
INCOV294758
INCOV294759
INCOV294760
INCOV294761
INCOV294762
INCOV294778
INCOV294822
INCOV294823
INCOV294824
INCOV294825
INCOV294826
INCOV294903
INCOV294904
INCOV294927
INCOV294999
INCOV295000
INCOV295241
INCOV295242
INCOV295290
INCOV295685
INCOV295691
INCOV295692
INCOV295996
INCOV295311
INCOV295312
INCOV295452
INCOV295453
INCOV295554
INCOV295555
INCOV295556
INCOV295557
INCOV295654
INCOV295779
INCOV295944
INCOV293903
INCOV294260
INCOV294297
INCOV294314
INCOV294316
INCOV294319
INCOV294320
INCOV294337
INCOV294338
INCOV294339
INCOV294340
INCOV294490
INCOV294498
INCOV294505
INCOV294558
INCOV294559
INCOV294560
INCOV294645
INCOV294791
INCOV294913
INCOV294986
INCOV295168
INCOV295169
INCOV295407
INCOV294763
INCOV294764
INCOV294765
INCOV294772
INCOV294779
INCOV294782
INCOV294827
INCOV294828
INCOV294856
INCOV294857
INCOV294858
INCOV294859
INCOV294905
INCOV294906
INCOV295253
INCOV295254
INCOV295255
INCOV295256
INCOV295705
INCOV295310
INCOV295468
INCOV295504
INCOV295558
INCOV295778
INCOV295780
INCOV295781
INCOV295921
INCOV295976
INCOV295907
INCOV294292
INCOV294293
INCOV294311
INCOV294312
INCOV294313
INCOV294315
INCOV294323
INCOV294341
INCOV294503
INCOV294561
INCOV294878
INCOV294879
INCOV294987
INCOV295007
INCOV295014
INCOV295107
INCOV295170
INCOV295171
INCOV295430
INCOV295442
INCOV295864
INCOV294773
INCOV294774
INCOV294780
INCOV294781
INCOV294783
INCOV294784
INCOV294829
INCOV294860
INCOV294861
INCOV294928
INCOV294929
INCOV295001
INCOV295002
INCOV295029
INCOV295257
INCOV295258
INCOV295259
INCOV295260
INCOV295522
INCOV295523
INCOV295524
INCOV295525
INCOV295526
INCOV295686
INCOV295693
INCOV295694
INCOV295717
INCOV295307
INCOV295308
INCOV295309
INCOV295469
INCOV295782
INCOV295840
INCOV295922
INCOV295923
INCOV295936
INCOV296033
INCOV295972
INCOV295982
INCOV296035
INCOV296039
INCOV294164
INCOV294310
INCOV294325
INCOV294326
INCOV294380
INCOV294491
INCOV294492
INCOV294493
INCOV294504
INCOV294529
INCOV294880
INCOV294881
INCOV295013
INCOV295017
INCOV295046
INCOV295047
INCOV295087
INCOV295431
INCOV295432
INCOV295443
INCOV294750
INCOV294766
INCOV294767
INCOV294768
INCOV294775
INCOV294785
INCOV294786
INCOV294808
INCOV294809
INCOV294830
INCOV294831
INCOV294907
INCOV295261
INCOV295276
INCOV295291
INCOV295472
INCOV295695
INCOV295696
INCOV295697
INCOV295698
INCOV295708
INCOV295925
INCOV295897
INCOV295898
INCOV295967
INCOV295983
INCOV296029
INCOV296032
INCOV295995
INCOV294261
INCOV294276
INCOV294306
INCOV294307
INCOV294308
INCOV294309
INCOV294342
INCOV294494
INCOV294495
INCOV294496
INCOV294497
INCOV294499
INCOV294500
INCOV294508
INCOV294562
INCOV294569
INCOV294570
INCOV295048
INCOV295049
INCOV295050
INCOV295051
INCOV295088
INCOV295177
INCOV294787
INCOV295263
INCOV295292
INCOV295687
INCOV295688
INCOV295305
INCOV295306
INCOV295454
INCOV295455
INCOV295470
INCOV295471
INCOV295630
INCOV295656
INCOV295984
INCOV296024
INCOV296034
INCOV294201
INCOV294343
INCOV294501
INCOV294502
INCOV294509
INCOV294510
INCOV294512
INCOV294563
INCOV294564
INCOV294565
INCOV294591
INCOV294882
INCOV295052
INCOV295053
INCOV295054
INCOV295055
INCOV295180
INCOV295181
INCOV295182
INCOV295406
INCOV295416
INCOV295433
INCOV295701
INCOV295865
INCOV295866
INCOV294751
INCOV294769
INCOV294770
INCOV294776
INCOV294777
INCOV294788
INCOV294789
INCOV294813
INCOV294815
INCOV294832
INCOV294833
INCOV294834
INCOV294862
INCOV294908
INCOV294909
INCOV294992
INCOV295030
INCOV295277
INCOV295293
INCOV295690
INCOV295820
INCOV295821
INCOV295977
INCOV296037
INCOV295993
INCOV294167
INCOV294168
INCOV294169
INCOV294225
INCOV294294
INCOV294324
INCOV294327
INCOV294507
INCOV294511
INCOV294566
INCOV294567
INCOV294571
INCOV294592
INCOV294610
INCOV294969
INCOV294989
INCOV295113
INCOV295114
INCOV295162
INCOV295183
INCOV295186
INCOV295413
INCOV295426
INCOV295434
INCOV295702
INCOV294930
INCOV294931
INCOV294932
INCOV295010
INCOV295262
INCOV295264
INCOV295265
INCOV295689
INCOV295456
INCOV295641
INCOV295642
INCOV295655
INCOV295783
INCOV295784
INCOV295785
INCOV295924
INCOV295952
INCOV295978
INCOV295994
INCOV294226
INCOV294227
INCOV294228
INCOV294229
INCOV294305
INCOV294513
INCOV294568
INCOV294970
INCOV294771
INCOV294810
INCOV294811
INCOV294812
INCOV294835
INCOV294836
INCOV294837
INCOV294838
INCOV294839
INCOV294840
INCOV294933
INCOV295011
INCOV295278
INCOV295279
INCOV295280
INCOV295281
INCOV295282
INCOV295283
INCOV295294
INCOV295295
INCOV295510
INCOV295511
INCOV295512
INCOV295513
INCOV295514
INCOV295515
INCOV295540
INCOV295541
INCOV295542
INCOV295543
INCOV295544
INCOV295545
INCOV295546
INCOV295547
INCOV295709
INCOV295801
INCOV295948
INCOV295949
INCOV295303
INCOV295304
INCOV295644
INCOV295770
INCOV295841
INCOV295968
INCOV295969
INCOV295970
INCOV296017
INCOV294262
INCOV294298
INCOV294301
INCOV294302
INCOV294303
INCOV294304
INCOV294514
INCOV294515
INCOV294530
INCOV294572
INCOV294578
INCOV294611
INCOV294914
INCOV295020
INCOV295108
INCOV295115
INCOV295116
INCOV295117
INCOV295187
INCOV295188
INCOV295190
INCOV295435
INCOV295794
INCOV295867
INCOV294793
INCOV294800
INCOV294801
INCOV294841
INCOV294842
INCOV294843
INCOV294863
INCOV294864
INCOV294865
INCOV294910
INCOV294911
INCOV294993
INCOV295004
INCOV295296
INCOV295394
INCOV295395
INCOV295396
INCOV295498
INCOV295527
INCOV295528
INCOV295529
INCOV295530
INCOV295531
INCOV295532
INCOV295533
INCOV295534
INCOV295535
INCOV295536
INCOV295537
INCOV295538
INCOV295539
INCOV295926
INCOV295950
INCOV295997
INCOV295457
INCOV295643
INCOV295809
INCOV295810
INCOV295851
INCOV295873
INCOV295874
INCOV295875
INCOV295880
INCOV295881
INCOV295945
INCOV295953
INCOV294263
INCOV294279
INCOV294328
INCOV294329
INCOV294344
INCOV294345
INCOV294518
INCOV294573
INCOV294574
INCOV294612
INCOV294613
INCOV294614
INCOV294875
INCOV294883
INCOV294884
INCOV294915
INCOV295123
INCOV295191
INCOV295193
INCOV295404
INCOV295412
INCOV295418
INCOV295425
INCOV295437
INCOV294794
INCOV294814
INCOV294844
INCOV294845
INCOV294912
INCOV295284
INCOV295297
INCOV295391
INCOV295392
INCOV295393
INCOV295568
INCOV295569
INCOV295570
INCOV295571
INCOV295572
INCOV295573
INCOV295574
INCOV295575
INCOV295699
INCOV295700
INCOV295831
INCOV295927
INCOV295602
INCOV295603
INCOV295604
INCOV295605
INCOV295606
INCOV295607
INCOV295608
INCOV295609
INCOV295610
INCOV295611
INCOV295612
INCOV295786
INCOV295937
INCOV295954
INCOV295979
INCOV296007
INCOV295971
INCOV296018
INCOV296019
INCOV296020
INCOV294163
INCOV294171
INCOV294230
INCOV294516
INCOV294519
INCOV294520
INCOV294916
INCOV295021
INCOV295101
INCOV295102
INCOV295103
INCOV295109
INCOV295163
INCOV295194
INCOV295195
INCOV295196
INCOV294405
INCOV294406
INCOV294700
INCOV294701
INCOV294795
INCOV294846
INCOV294848
INCOV294849
INCOV294850
INCOV294851
INCOV294852
INCOV294975
INCOV294994
INCOV295286
INCOV295298
INCOV295299
INCOV295302
INCOV295387
INCOV295388
INCOV295389
INCOV295390
INCOV295499
INCOV295560
INCOV295561
INCOV295562
INCOV295563
INCOV295564
INCOV295565
INCOV295566
INCOV295567
INCOV295711
INCOV295712
INCOV295720
INCOV295721
INCOV295799
INCOV295832
INCOV295928
INCOV296030
INCOV296031
INCOV296038
INCOV294170
INCOV294300
INCOV294330
INCOV294346
INCOV294347
INCOV294575
INCOV294579
INCOV294615
INCOV295164
INCOV295165
INCOV295243
INCOV295403
INCOV295441
INCOV294396
INCOV294397
INCOV294398
INCOV294399
INCOV294400
INCOV294401
INCOV294407
INCOV294408
INCOV294702
INCOV294703
INCOV294802
INCOV294805
INCOV295288
INCOV295300
INCOV295383
INCOV295384
INCOV295385
INCOV295386
INCOV295583
INCOV295584
INCOV295585
INCOV295586
INCOV295587
INCOV295588
INCOV295589
INCOV295590
INCOV295591
INCOV295592
INCOV295593
INCOV295594
INCOV295595
INCOV295744
INCOV295765
INCOV295766
INCOV295929
INCOV295552
INCOV295553
INCOV295645
INCOV295811
INCOV295882
INCOV295883
INCOV294264
INCOV294265
INCOV294299
INCOV294348
INCOV294349
INCOV294517
INCOV294521
INCOV294522
INCOV294531
INCOV294576
INCOV294577
INCOV294580
INCOV294581
INCOV294585
INCOV294586
INCOV294593
INCOV294616
INCOV294918
INCOV295420
INCOV295507
INCOV294704
INCOV294796
INCOV294797
INCOV294818
INCOV294934
INCOV294935
INCOV294995
INCOV294996
INCOV295285
INCOV295379
INCOV295380
INCOV295381
INCOV295382
INCOV295713
INCOV295714
INCOV295715
INCOV295731
INCOV295732
INCOV295930
INCOV295458
INCOV295459
INCOV295646
INCOV295647
INCOV295842
INCOV295884
INCOV295955
INCOV295956
INCOV295957
INCOV296021
INCOV296022
INCOV296026
INCOV294202
INCOV294317
INCOV294321
INCOV294322
INCOV294331
INCOV294332
INCOV294523
INCOV294526
INCOV294527
INCOV294528
INCOV294594
INCOV294617
INCOV294618
INCOV294919
INCOV294971
INCOV295015
INCOV295244
INCOV295436
INCOV294804
INCOV295733
INCOV295745
INCOV295648
INCOV295807
INCOV295808
INCOV295852
INCOV292233
INCOV294318
INCOV294524
INCOV294532
INCOV294798
INCOV294799
INCOV294866
INCOV294936
INCOV294937
INCOV294997
INCOV295287
INCOV295376
INCOV295377
INCOV295378
INCOV295576
INCOV295577
INCOV295578
INCOV295579
INCOV295580
INCOV295581
INCOV295582
INCOV295724
INCOV295725
INCOV295726
INCOV295727
INCOV295730
INCOV295734
INCOV295735
INCOV295736
INCOV295460
INCOV295461
INCOV295548
INCOV295549
INCOV295550
INCOV295551
INCOV295559
INCOV295755
INCOV295818
INCOV295819
INCOV295843
INCOV295876
INCOV295877
INCOV295938
INCOV295985
INCOV296002
INCOV294266
INCOV294362
INCOV294525
INCOV294533
INCOV294534
INCOV294535
INCOV294582
INCOV294583
INCOV294584
INCOV294587
INCOV294604
INCOV294619
INCOV294620
INCOV294621
INCOV294622
INCOV294623
INCOV294718
INCOV294885
INCOV294920
INCOV294921
INCOV295093
INCOV295094
INCOV295095
INCOV295096
INCOV295097
INCOV295104
INCOV295657
INCOV295795
INCOV294816
INCOV294817
INCOV294819
INCOV294876
INCOV294939
INCOV294940
INCOV295289
INCOV295301
INCOV295370
INCOV295371
INCOV295372
INCOV295373
INCOV295374
INCOV295375
INCOV295500
INCOV295501
INCOV295722
INCOV295723
INCOV295728
INCOV295729
INCOV295737
INCOV295746
INCOV295747
INCOV295748
INCOV295749
INCOV295750
INCOV295823
INCOV295845
INCOV295846
INCOV295899
INCOV295900
INCOV294152
INCOV294203
INCOV294267
INCOV294268
INCOV294474
INCOV294595
INCOV294597
INCOV294624
INCOV294625
INCOV294626
INCOV294719
INCOV294955
INCOV294956
INCOV294957
INCOV294958
INCOV294959
INCOV294960
INCOV294961
INCOV294962
INCOV294963
INCOV294964
INCOV294965
INCOV294966
INCOV294967
INCOV294972
INCOV295008
INCOV295022
INCOV295098
INCOV295099
INCOV295100
INCOV295110
INCOV295111
INCOV295198
INCOV295272
INCOV295417
INCOV295658
INCOV295868
INCOV294941
INCOV294942
INCOV295362
INCOV295363
INCOV295364
INCOV295365
INCOV295366
INCOV295367
INCOV295368
INCOV295369
INCOV295473
INCOV295624
INCOV295738
INCOV295741
INCOV295742
INCOV295751
INCOV295752
INCOV295753
INCOV295754
INCOV295802
INCOV295931
INCOV295817
INCOV295822
INCOV295828
INCOV295844
INCOV295878
INCOV295879
INCOV295901
INCOV295902
INCOV295946
INCOV296008
INCOV295986
INCOV296040
INCOV294277
INCOV294280
INCOV294333
INCOV294334
INCOV294596
INCOV294598
INCOV294599
INCOV294600
INCOV294605
INCOV294606
INCOV294720
INCOV294792
INCOV294922
INCOV294923
INCOV294924
INCOV295009
INCOV295203
INCOV295245
INCOV295246
INCOV295401
INCOV295402
INCOV295421
INCOV295703
INCOV295718
INCOV294820
INCOV294847
INCOV294943
INCOV294944
INCOV294945
INCOV294998
INCOV295005
INCOV295358
INCOV295359
INCOV295360
INCOV295361
INCOV295596
INCOV295597
INCOV295598
INCOV295599
INCOV295600
INCOV295601
INCOV295623
INCOV295627
INCOV295739
INCOV295740
INCOV295743
INCOV295824
INCOV295833
INCOV295932
INCOV295848
INCOV296025
INCOV294153
INCOV294231
INCOV294232
INCOV294233
INCOV294234
INCOV294235
INCOV294236
INCOV294237
INCOV294238
INCOV294239
INCOV294240
INCOV294241
INCOV294242
INCOV294248
INCOV294269
INCOV294361
INCOV294536
INCOV294601
INCOV294602
INCOV294603
INCOV294627
INCOV294721
INCOV294886
INCOV294887
INCOV294973
INCOV295092
INCOV295105
INCOV295106
INCOV295213
INCOV295357
INCOV295509
INCOV295710
INCOV295006
INCOV295353
INCOV295354
INCOV295355
INCOV295356
INCOV295625
INCOV295626
INCOV295756
INCOV295757
INCOV295767
INCOV295462
INCOV295463
INCOV295464
INCOV295813
INCOV295903
INCOV295958
INCOV295959
INCOV295960
INCOV295961
INCOV296009
INCOV296010
INCOV296003
INCOV294360
INCOV294607
INCOV294608
INCOV294628
INCOV294629
INCOV294630
INCOV294710
INCOV294888
INCOV294889
INCOV294894
INCOV294974
INCOV295205
INCOV295206
INCOV295207
INCOV295214
INCOV295506
INCOV295508
INCOV295664
INCOV295707
INCOV294946
INCOV294947
INCOV294948
INCOV294949
INCOV295346
INCOV295347
INCOV295348
INCOV295349
INCOV295350
INCOV295351
INCOV295352
INCOV295466
INCOV295474
INCOV295502
INCOV295622
INCOV295628
INCOV295772
INCOV295773
INCOV295825
INCOV295834
INCOV295836
INCOV295933
INCOV295934
INCOV295465
INCOV295849
INCOV295893
INCOV295895
INCOV295939
INCOV295940
INCOV295941
INCOV296000
INCOV296023
INCOV294147
INCOV294148
INCOV294149
INCOV294150
INCOV294151
INCOV294249
INCOV294270
INCOV294609
INCOV294711
INCOV294712
INCOV294713
INCOV294715
INCOV294716
INCOV294722
INCOV295208
INCOV295215
INCOV295247
INCOV295422
INCOV295659
INCOV295660
INCOV295662
INCOV295665
INCOV295666
INCOV295719
INCOV295343
INCOV295344
INCOV295345
INCOV295758
INCOV295759
INCOV295814
INCOV295850
INCOV295962
INCOV295980
INCOV296027
INCOV296028
INCOV294243
INCOV294244
INCOV294245
INCOV294246
INCOV294247
INCOV294717
INCOV294723
INCOV295023
INCOV295027
INCOV295706
INCOV294803
INCOV294807
INCOV295341
INCOV295342
INCOV295629
INCOV295835
INCOV295812
INCOV295815
INCOV295847
INCOV295885
INCOV295886
INCOV295892
INCOV295965
INCOV295966
INCOV296001
INCOV294154
INCOV294271
INCOV294272
INCOV294335
INCOV294336
INCOV294403
INCOV294714
INCOV294730
INCOV294731
INCOV294890
INCOV294891
INCOV294926
INCOV295025
INCOV295211
INCOV295216
INCOV295217
INCOV295266
INCOV295267
INCOV295340
INCOV295423
INCOV295661
INCOV295796
INCOV294806
INCOV294950
INCOV294951
INCOV294952
INCOV294953
INCOV295339
INCOV295444
INCOV295760
INCOV295761
INCOV295774
INCOV295803
INCOV295826
INCOV295870
INCOV295871
INCOV295935
INCOV295973
INCOV295998
INCOV295816
INCOV295827
INCOV295981
INCOV294250
INCOV294251
INCOV294252
INCOV294273
INCOV294726
INCOV294727
INCOV294892
INCOV294893
INCOV295026
INCOV295212
INCOV295218
INCOV295219
INCOV295220
INCOV295221
INCOV295222
INCOV295223
INCOV295224
INCOV295225
INCOV295226
INCOV295270
INCOV295271
INCOV295273
INCOV295663
INCOV295667
INCOV295668
INCOV295704
INCOV295797
INCOV295798
INCOV295329
INCOV295330
INCOV295331
INCOV295332
INCOV295333
INCOV295334
INCOV295335
INCOV295336
INCOV295337
INCOV295338
INCOV295445
INCOV295446
INCOV295447
INCOV295649
INCOV295650
INCOV295762
INCOV295768
INCOV295910
INCOV295829
INCOV295887
INCOV296011
INCOV296012
INCOV296036
INCOV294274
INCOV294386
INCOV294387
INCOV294388
INCOV294389
INCOV294390
INCOV294391
INCOV294392
INCOV294393
INCOV294394
INCOV294395
INCOV294724
INCOV294725
INCOV294728
INCOV294729
INCOV294732
INCOV294733
INCOV294734
INCOV294754
INCOV294755
INCOV295024
INCOV295231
INCOV295268
INCOV295269
INCOV295495
INCOV295669
INCOV295670
INCOV295671
INCOV295677
INCOV295448
INCOV295467
INCOV295503
INCOV295631
INCOV295632
INCOV295633
INCOV295634
INCOV295804
INCOV295805
INCOV295806
INCOV295837
INCOV295911
INCOV295912
INCOV295913
INCOV295914
INCOV295915
INCOV295987
INCOV295989
INCOV296004
INCOV294204
INCOV294281
INCOV294404
INCOV294705
INCOV294736
INCOV294747
INCOV294752
INCOV294753
INCOV294895
INCOV294896
INCOV295028
INCOV295228
INCOV295232
INCOV295274
INCOV295328
INCOV295516
INCOV295517
INCOV295518
INCOV295519
INCOV295520
INCOV295521
INCOV295675
INCOV295676
INCOV295325
INCOV295326
INCOV295327
INCOV295838
INCOV295974
INCOV295830
INCOV295894
INCOV295904
INCOV295908
INCOV295963
INCOV295964
INCOV295988
INCOV296005
INCOV296006
INCOV295323
INCOV295324
INCOV295763
INCOV295764
INCOV295771
INCOV295872
INCOV295916
INCOV295888
INCOV295905
INCOV295942
INCOV296013
INCOV296014
INCOV295839

**Table 5:** Questionnaire template used for the telephonic interview

Demographic Data	
1. Sample ID	
2. Patient Name	
3. Gender	
4. Age (in years)	
5. Phone No	
6. District of Residence	
7. Sample collection date	
8. SARS-CoV-2 Variant	
Clinical Data	
1. Presence of Symptoms	1. Present 2. Absent
a. Fever	1. Yes 2. No
b. Cold	1. Yes 2. No
c. Cough	1. Yes 2. No
d. Sore throat	1. Yes 2. No
e. Breathlessness	1. Yes 2. No
f. Headache	1. Yes 2. No
g. Runny Nose	1. Yes 2. No
h. Weakness	1. Yes 2. No
i. Myalgia	1. Yes 2. No
j. Body ache	1. Yes 2. No
k. Vomiting	1. Yes 2. No
l. Diarrhea	1. Yes 2. No
m. Loss of Taste	1. Yes 2. No
n. Loss of Smell	1. Yes 2. No
o. Other Symptoms (If Any)	
2. Presence of Co-morbidities	1. Present 2. Absent
What Co-morbidities present	
3. Home isolation	1. Yes 2. No
4. Hospitalization/ Institutional Quarantine	1. Yes 2. No
5. Oxygen requirement	1. Yes 2. No
a. Non-Invasive	1. Yes 2. No
b. Invasive (Intubation)	1. Yes 2. No
6. History of Previous Infection	1. Yes 2. No
7. Travel history	1. Yes 2. No
8. Vaccine Taken	1. Yes 2. No 3. Not Applicable
9. Vaccine name	1. Covishield 2. Covaxin 3. Pfizer 4. Others
a. Dose 1 Taken	1. Yes 2. No
b. Dose 2 Taken	1. Yes 2. No
c. Booster (Precautionary) Dose Taken	1. Yes 2. No
10. Type of Treatment received?	1. Symptomatic (Antipyretics, multivitamins, antihistaminic) 2. Oxygen therapy 3. Steroids 4. Antivirals 5. Monoclonal Antibodies 6. Others (Specify)
11. Outcome	1. Alive 2. Dead
